# A Strategy for Efficient Preparation of Genus-Specific Diagnostic Antibodies for Snakebites

**DOI:** 10.3389/fimmu.2021.775678

**Published:** 2021-11-09

**Authors:** Chengbo Long, Feilong Wu, Qiumin Lu, Bing Xie, Chuanbin Shen, Jiayao Li, Yanling Deng, Ping Liang, Yongzhi Yu, Ren Lai

**Affiliations:** ^1^ Key Laboratory of Animal Models and Human Disease Mechanisms, Key Laboratory of Bioactive Peptides of Yunnan Province, Engineering Laboratory of Bioactive Peptides, The National & Local Joint Engineering Center of Natural bioactive Peptides, Kunming Institute of Zoology-The Chinese University of Hong Kong (KIZ-CUHK) Joint Laboratory of Bioresources and Molecular Research in Common Diseases, National Resource Center for Non-Human Primates, Kunming Primate Research Center, and National Research Facility for Phenotypic & Genetic Analysis of Model Animals (Primate Facility), Kunming Institute of Zoology, Chinese Academy of Sciences, Kunming, China; ^2^ Kunming College of Life Science, University of Chinese Academy of Sciences, Kunming, China; ^3^ Sino-Africa Joint Research Center, Chinese Academy of Sciences, Wuhan, China; ^4^ Institute of Biology Leiden, Leiden University, Leiden, Netherlands; ^5^ Department of Laboratory Medicine, Li Ka Shing Knowledge Institute (LKSKI)-Keenan Research Centre for Biomedical Science, St. Michael’s Hospital, University of Toronto, Toronto, ON, Canada; ^6^ Clinical Laboratory, Hospital of Traditional Chinese Medicine of Wuzhou, Wuzhou, China

**Keywords:** snakebite envenoming, venom, antigen, immunogenicity, diagnosis

## Abstract

As said by former United Nations Secretary-General Kofi Annan, “Snakebite is the most important tropical disease you’ve never heard of.” Listed as a priority neglected tropical disease by the World Health Organization, snakebite envenoming (SBE) kills in excess of 125,000 people per year. However, due to the complexity and overlap of snake venom compositions, few reliable venom diagnostic methods for genus-/species-specific identification, which is crucial for successful SBE therapy, are available. Here, we develop a strategy to select and prepare genus-specific snake venom antibodies, which allows rapid and efficient clinical diagnosis of snakebite. Multi-omics approaches are used to choose candidate antigens from snake venoms and identify genus-specific antigenic epitope peptide fragments (GSAEPs) with ideal immunogenicity, specificity, and spatial accessibility. Double-antibody sandwich ELISA kit was established by matching a polyclonal antibody against a natural antigen and a monoclonal antibody that was prepared by natural protein as antigen and can specifically target the GSAEPs. The kit shows the ability to accurately identify venoms from similar genera of *Trimeresurus* and *Protobothrops* with a detection limit of 6.25 ng/ml on the snake venoms and a little cross-reaction, thus proving high feasibility and applicability.

## Introduction

There are about 3,596 species of snakes worldwide and 768 of them are venomous. Elapidae (elapids, consisting of nearly 300 species and 60 genera) and Viperidae (vipers, 329 species and 27 genera) are two strictly venomous families. Some species from the families of Colubridae (colubrids) and Lamprophiidae (lamprophiids) are also venomous, although most of the members are non-venomous. Nevertheless, most of the lethal cases are almost exclusively resulting from snakebite envenoming (SBE) of Viperidae and Elapidae (1, 2). In tropical and subtropical countries, SBE is a neglected public health issue and often leads to life-threatening conditions. There are about 5.4 million snakebites annually, which results in 1.8 to 2.7 million SBE cases and at least 125,000 deaths (3). Due to many incidences occurring in rural areas with poor health and transportation facilities, it is difficult to accurately estimate the global rates of snakebite and associated mortality, thus likely underreporting worldwide estimates.

Snake venom is a mixture of toxins. Major toxic components of snake venoms are peptides and proteins, which include four dominant protein families, namely, phospholipase A_2_, metalloproteases, serine proteases, and three-finger toxins; six secondary protein families, namely, cysteine-rich secretory proteins, *L*-amino acid oxidases, Kunitz peptides, C-type lectins, disintegrins, and natriuretic peptides; and at least 36 rarer protein families (4). Most of SBE results from the dominant and secondary protein families, which form the bulk of snake venoms. These toxins are highly consistent within genera but are quite different among different genera (5–9). Due to the heterogeneity of venom composition, accurate diagnosis of snakebite is essential for envenomation treatment. Identifying the offending snake genus or type of venom efficiently facilitates clinicians to predict and prepare for possible clinical manifestations, especially in antivenom treatment. Even today, many snakebite diagnoses have relied on detailed clinical symptomatology or description of the offending snake by the patient or other witnesses (10). However, identification of different groups of snakes, whose venom components likely exhibit extensive cross-reactivity, is a challenging task. Therefore, scientists continue in their attempts to solve this problem by developing specific snake venom diagnostic techniques including enzymatic activity and forensic genetic analyses but primarily involving immunoassays [e.g., enzyme-linked immunosorbent assay (ELISA)] and constituent antibodies. However, although experimental diagnosis is increasingly developed, many of the limits in detection specificity remain unresolved (11). Thus, the development of genus-specific antiserum and diagnostic methods has become a new trend in the diagnosis of snakebites and SBE treatment.

The application of specific antibodies in the diagnosis of snakebite can accurately judge the type of snakebite and prevent unwarranted and wasteful administration of antivenom (11). To date, most of the diagnostic antibodies were polyclonal antibodies with poor specificity and often saturate at a high concentration of snake venom, for example, the polyclonal diagnostic antibodies for distinguishing hematotoxin and neurotoxin and the polyclonal diagnostic antibodies for individual snake species (12), while the contents of snake venom in the plasma of different patients, bitten by snakes of the same species, vary up to 100-folds (from a few nanograms to hundreds of nanograms) (12, 13). Although monoclonal antibodies have been reported, they cannot be used in the clinic because of their low specificity (14, 15). At present, there is no reliable antibody for diagnosis, resulting in the low accuracy of clinical diagnosis, which hinders the selection and dosage of antiserum.

**Figure 1:**
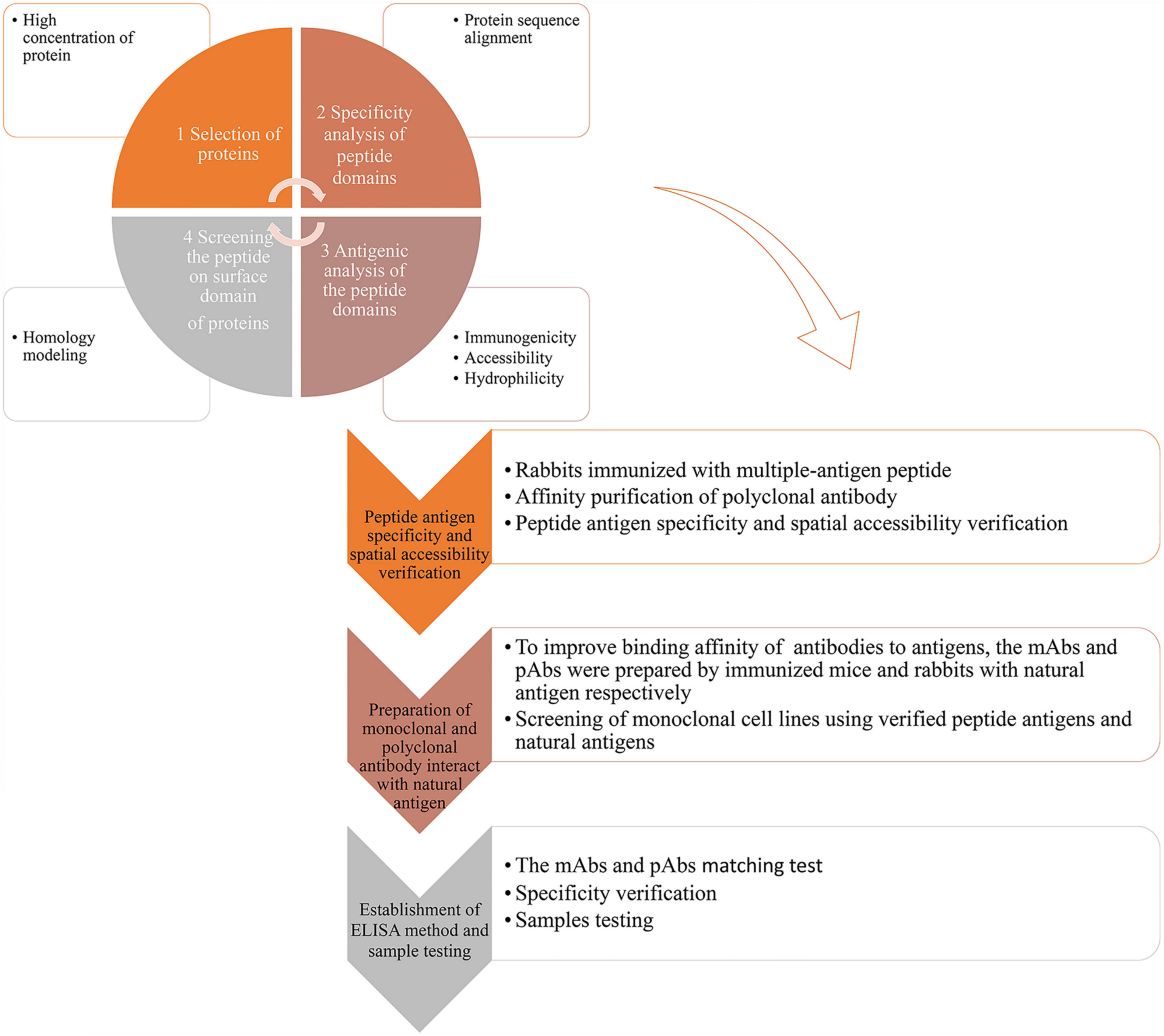
Graphical abstract of the antibody development strategy. 1) Based on data mining and analysis of snake genomes, transcriptomes, and proteomes, highly abundant proteins of representing species of a genus were selected. Specific peptides of the representing species were identified by BLAST analysis. Using the Protean module in Lasergene, the antigenic properties of the protein were analyzed, and antigenicity of the specific peptide was determined, including immunogenicity, accessibility, secondary structure, and hydrophilicity. Based on the protein structure or homology modeling results in SWISS-MODEL (online), the spatial position of specific peptides in the protein was confirmed, and specific peptides located on the surface of the protein were selected. 2) pAbs-p against peptide antigens were prepared by immunizing rabbits with KLH–peptide complex antigens. Antigen binding ability, antigen specificity, and accessibility of pAbs-p were verified. 3) Natural protein antigens containing peptide antigens were isolated and purified, and mAbs-n and pAbs-n against protein antigens were prepared. Binding ability and specificity of mAbs-n and pAbs-n were detected. 4) mAbs-n and pAbs-n were matched and verified by ELISA. Apparent concentrations of snake venom using simulated snakebites were detected.

In this work, we propose a strategy for the efficient preparation of genus-specific diagnostic antibodies for snakebites, as follows: 1) data mining and multi-omics analysis of snake venom glands were performed to obtain information on proteins in different snake venoms and select potential antigens, which are highly abundant proteins containing ideal antigenic epitopes in various genera; 2) antigenic epitope peptide fragments, whose immunogenicity, specificity, and spatial accessibility are validated and verified, and natural venomous proteins containing the antigenic epitopes are used for the preparation of antibodies; and 3) the antibodies were used for the establishment of double-antibody sandwich ELISA to identify and verify venoms from different snakes (**Figure 1**). Based on the strategy, we successfully developed diagnostic antibodies against the *Trimeresurus* and *Protobothrops* genera, which are a sister clade to the New World pit vipers and belong to the top 10 venomous snakes in China. Most of them are distributed in South China and resulted in hundred thousands of snakebites each year in China, with the most clinical significance.

## Results

### Antigen Analysis and Preparation of pAbs Against Peptide Antigens

**Figure 2:**
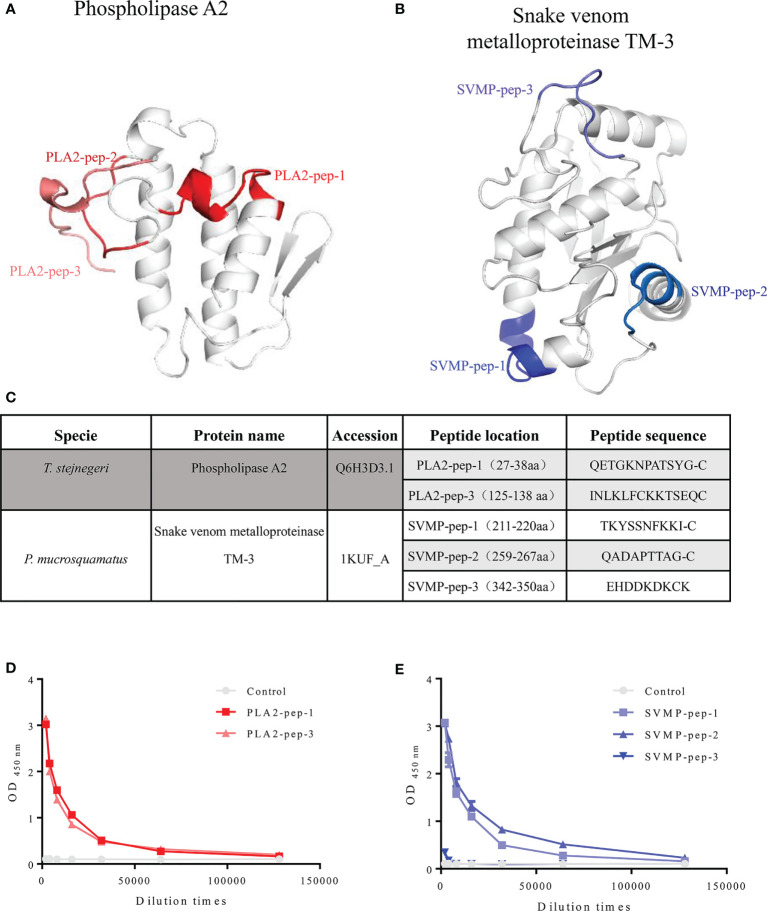
Information on peptide antigens. Structures of phospholipase A_2_ (access: Q6H3D3.1) and snake venom metalloproteinase TM-3 (access: 1kuf) of *T. stejnegeri* and *P. mucrosquamatus*, respectively, were obtained from the Protein Data Bank. After analysis of amino acid sequences, antigen immunogenicity, and three-dimensional structure of phospholipase A_2_ and snake venom metalloproteinase TM-3, respectively, the genus-specific antigenic peptides of phospholipase A_2_ and snake venom metalloproteinase TM-3 were obtained. Three peptide antigens of PLA_2_ (i.e., PLA2-pep-1, PLA2-pep-2, and PLA2-pep-3) and three peptide antigens of SVMP (i.e., SVMP-pep-1, SVMP-pep-2, and SVMP-pep-3) were obtained. Peptide PLA2-pep-2 was shielded by PLA2-pep-1, and thus, antigen PLA2-pep-2 was discarded **(A**, **B)**. Peptide antigens were synthesized, and cysteine was added to the peptide sequence for KLH–peptide complex antigen preparation if the chosen peptides did not contain cysteine at the C-terminal **(C)**. Based on ELISA of peptide antigen-coated plates, the titers of purified pAbs-p (1 mg/ml) against PLA2-pep-1, PLA2-pep-3, SVMP-pep-1, and SVMP-pep-2 were 1:64,000 or more, while the titers of anti-SVMP-pep-3 were only 1:1,000 **(D**, **E)**.

After screening available venom gland transcriptome and proteome data, we selected phospholipase A_2_ (PLA_2_, UniProt access: Q6H3D3.1) and snake venom metalloproteinase TM-3 (SVMP, UniProt access: 1KUF_-_A) in *Trimeresurus stejnegeri* and *Protobothrops mucrosquamatus*, respectively, as the primary antigens due to their high content (16, 17). The amino acid sequences of PLA_2_ and SVMP were analyzed by a BLAST search of the NCBI database to identify genus-specific peptide antigens (**Supplementary Figures 1, 2**). Combining antigen immunogenicity (**Supplementary Figures 3, 4**) and structural analyses (**Supplementary Figures 5, 6**), several genus-specific antigenic peptides of PLA_2_ and SVMP were designed including three peptide antigens for PLA_2_ (PLA2-pep-1, PLA2-pep-2, and PLA2-pep-3) (**Figure 2A**) and three peptide antigens for SVMP (SVMP-pep-1, SVMP-pep-2, and SVMP-pep-3) (**Figure 2B**). Structural modeling suggests that PLA-pep-2 was shielded by PLA2-pep-3, and thus, PLA-pep-2 was discarded. The above peptide antigens were synthesized. If the original peptides did not contain cysteine, a single cysteine was added to the peptide at the C-terminal for the preparation of a keyhole limpet hemocyanin (KLH)–peptide complex antigen (**Figure 2C**). The synthetic peptide antigens were cross-linked to KLH, and two rabbits were immunized using each KLH–peptide complex antigen. The plasma of the rabbit with a higher antibody titer was selected for affinity antigen purification and antigen accessibility and specificity evaluation. Using ELISA coated with peptide antigen, the titers of the purified polyclonal antibodies (called pAbs-p, concentration was 1 mg/ml) against PLA2-pep-1, PLA2-pep-3, SVMP-pep-1, and SVMP-pep-2 were more than 1:64,000, while the titers of pAbs-p against SVMP-pep-3 were only 1:1,000 (**Figures 2D, E** **)**.

### Evaluation of Peptide Antigen Specificity and Accessibility

The top 10 venomous snakes in China are from the Elapidae family (*Naja atra*, *Bungarus fasciatus*, *Bungarus multicinctus*, *Ophiophagus hannah*, and *Hydrophis cyanocinctus*), Viperinae subfamily (*Daboia russelii siamensis*), and Crotalinae subfamily (*Gloydius blomhoffi siniticus*, *T. stejnegeri*, *Deinagkistrodon acutus*, and *P. mucrosquamatus*). To verify the specificity of the peptide antigens, *N. atra* and *D. r. siamensis* were selected as representing species of the Elapidae family and Viperinae subfamily, respectively, and *D. acutus*, *T. stejnegeri*, and *P. mucrosquamatus* were selected as representing species of the Crotalinae subfamily. Crude venom samples from these five snake species were electrophoresed under reducing conditions and stained with Coomassie Brilliant Blue G-250 (**Supplementary Figure 7**). Results showed that all the venoms from Viperinae and Crotalinae subfamilies shared a similar protein profile and their major components ranged in size from 15 to 100 kDa. Venom from the Elapidae family contained a greater amount of low-molecular weight proteins that ranged in size from 10 to 20 kDa. These findings are consistent with those reported in previous research (9).

**Figure 3:**
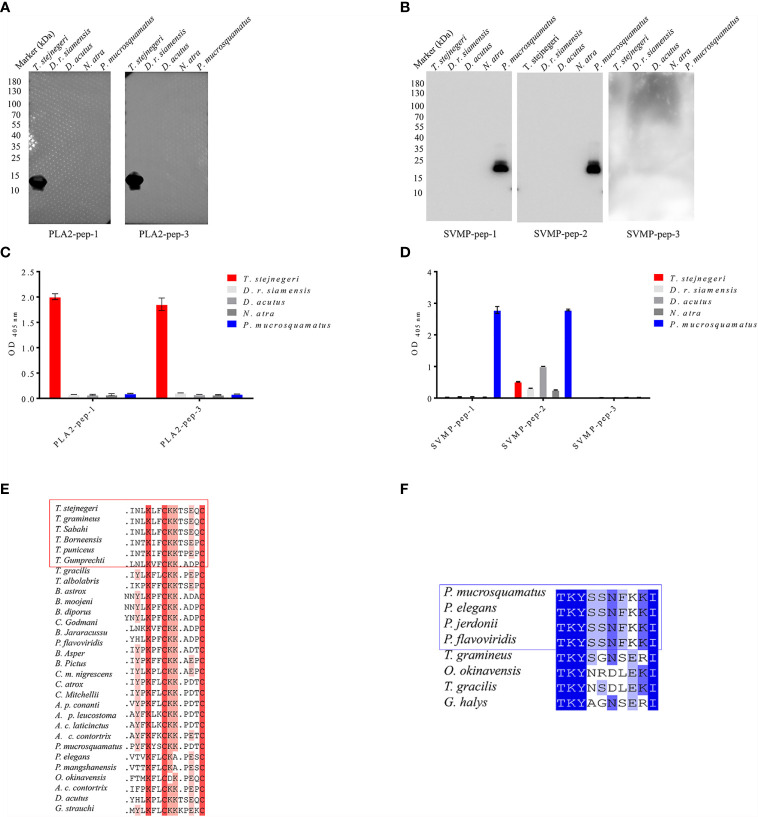
Accessibility and specificity of peptide antigens. Based on Western blot analysis, anti-PLA2-pep-1, anti-PLA2-pep-3, anti-SVMP-pep-1, and anti-SVMP-pep-2 antibodies were specific to the venom of five species (*T. stejnegeri*, *D. r. siamensis*, *D. acutus*, *N. atra*, and *P. mucrosquamatus*) and showed single bands, while anti-SVMP-pep-3 showed no target band **(A**, **B)**. Antigen accessibility of peptide antigens in natural protein was determined based on ELISA of venom-coated plates. Results showed that PLA2-pep-1, PLA2-pep-3, SVMP-pep-1, and SVMP-pep-2 were surface accessible. Moreover, PLA2-pep-1, PLA2-pep-3, and SVMP-pep-1 showed species specificity, but SVMP-pep-2 showed low species specificity and cross-reactions with the other four snake venoms **(C**, **D)**. The sequence blast analysis of PLA2-pep-3 and SVMP-pep-1 showed that PLA2-pep-3 and SVMP-pep-1 have the best specificity in genus *Trimeresurus* and *Protobothrops*, respectively **(E, F)**.

The specificity of pAbs-p prepared by peptide as antigens in the venom of five snake species was verified by Western blot analysis. Results showed that against PLA2-pep-1, PLA2-pep-3, SVMP-pep-1, and SVMP-pep-2 antibodies, which showed single bands, were specific for their corresponding polypeptide antigens and snake species, whereas against SVMP-pep-3 antibodies showed no detectable band (**Figures 3A, B** **)**. Based on ELISA of venom-coated plates, the antigen accessibility of the peptides in the natural protein was detected, indicating that PLA2-pep-1, PLA2-pep-3, SVMP-pep-1, and SVMP-pep-2 were surface accessible (**Figure 3D**). The specificity of the antibodies against the peptide antigens in the venom of different snakes was verified by ELISA. As illustrated in **Figures 3C, D**, against PLA2-pep-1, PLA2-pep-3, and SVMP-pep-1 antibodies were species-specific, while against SVMP-pep-2 antibodies showed low species specificity and cross-reaction with the venom of the other four snake species. The sequence analysis of these antigen peptides showed that the sequences of PLA2-pep-3 and SVMP-pep-1 have the best specificity in *Trimeresurus* PLA_2_ and *Protobothrops* SVMP, although the two families of proteins are widely distributed in viper venoms (**Figures 3E, F** **)**. Based on the results, PLA2-pep-3 and SVMP-pep-1 qualified as antigens.

### Preparation of mAbs and pAbs Against Natural Antigens

**Figure 4:**
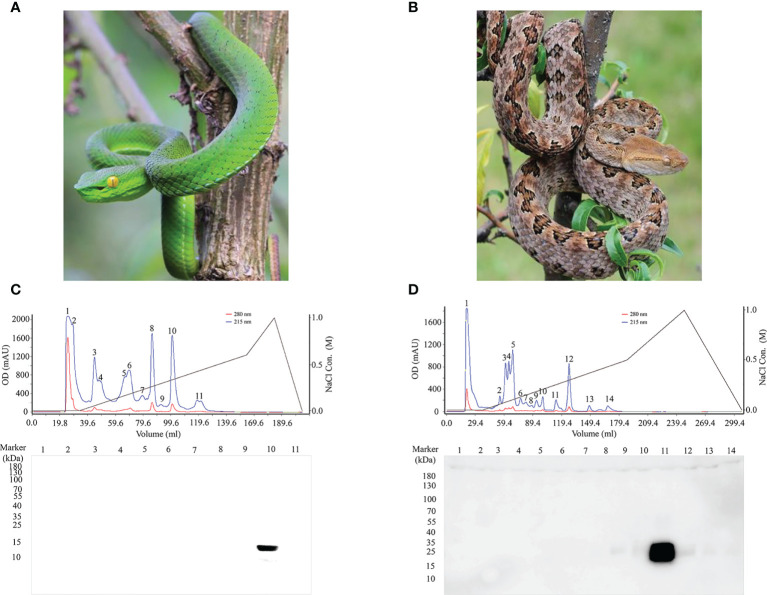
Purification of natural antigens of PLA_2_ and SVMP from *T. stejnegeri* and *P. mucrosquamatus*, respectively. Using FPLC, PLA_2_ and SVMP were purified from crude *T. stejnegeri* **(A)** and *P. mucrosquamatus* venom **(B)**, respectively (photographs were provided by Ma Xiaofeng). Crude venom (50 mg) of *T. stejnegeri* and *P. mucrosquamatus* was dissolved in 1 ml of phosphate buffer, loaded into the FPLC system, and eluted with a sodium chloride gradient. 11 [**(C)**, upper] and 14 fractions [**(D)**, upper] were obtained from crude *T. stejnegeri* and *P. mucrosquamatus* venom, respectively. SDS-PAGE and Western blotting with anti-PLA2-pep-3 and anti-SVMP-pep-1 pAbs-p were used to detect the fractions. Results showed that the 10th fraction was the target PLA_2_ protein in *T. stejnegeri* venom [**(C)**, lower] and the 11th fraction was the target SVMP protein in *P. mucrosquamatus* [**(D)**, lower].

To obtain natural antigens, PLA_2_ and SVMP were purified from crude *T. stejnegeri* (**Figure 4A**) and *P. mucrosquamatus* (**Figure 4B**) venom using fast protein liquid chromatography (FPLC) with a Resource™ S column (6 ml). Crude venom (50 mg) from *T. stejnegeri* and *P. mucrosquamatus* was dissolved in 1 ml of phosphate buffer and then loaded into the FPLC system and eluted with a sodium chloride gradient, respectively. Crude venom from *T. stejnegeri* and *P. mucrosquamatus* was separated into 11 (**Figure 4C**, upper) and 14 fractions (**Figure 4D**, upper), respectively. Sodium dodecyl sulfate-polyacrylamide gel electrophoresis (SDS-PAGE) and Western blot analysis with anti-PLA2-pep-3 or anti-SVMP-pep-1 pAbs-p were used to detect the fractions. Results showed that the 10th fraction was the PLA_2_ protein in the *T. stejnegeri* venom (**Figure 4C**, lower) and the 11th fraction was the SVMP protein in the *P. mucrosquamatus* venom (**Figure 4D**, lower). The target fractions were analyzed by SDS-PAGE. The PLA_2_ fraction was a pure protein, while the SVMP fraction had two protein bands, namely, SVMP and other protein (**Supplementary Figure 8**).

**Figure 5:**
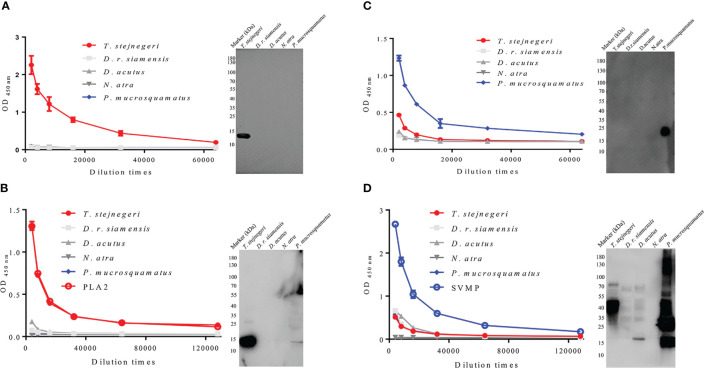
Antigen specificity of mAbs-n and pAbs-n from mice and rabbits. mAbs-n and pAbs-n against natural PLA_2_ and SVMP protein antigens were prepared by immunizing mice and rabbits with natural PLA_2_ and SVMP protein antigens, respectively. Specificity and accessibility of PLA2-pep-3 were verified by mAbs-n (PLA2-mc8) **(A)**. Specificity and accessibility of SVMP-pep-1 were verified by mAbs-n (SVMP-mc4) **(C)**. The pAb titers of anti-PLA_2_ and anti-SVMP antibodies determined by ELISA were higher than 1:128,000. ELISA and Western blotting showed that anti-PLA_2_ pAbs-n had good specificity, while anti-SVMP pAbs-n had lower specificity with obvious cross-reaction with *T. stejnegeri*, *D. r. siamensis*, and *D. acutus* venom **(B**, **D)**.

Compared with peptide antigens, the binding affinity of anti-peptide antigen polyclonal antibodies to natural protein antigens (and vice versa) is weak (**Supplementary Figures 9, 10**). To improve the binding affinity of antibodies to natural antigens, the monoclonal antibodies (mAbs) and pAbs (called mAbs-n and pAbs-n correspondingly) against the natural PLA_2_ and SVMP protein antigens were prepared by immunizing mice and rabbits with the purified natural antigens, respectively. In the preparation of mAbs-n, the spleen cells of immunized mice were fused with SP2/0 cells (mouse myeloma cells). Positive clones against the natural antigens were screened out (**Supplementary Figures 11A, C**). Three ideal cell lines, which secreted mAb with high affinity to PLA2-pep-3 and PLA_2_, were obtained (**Supplementary Figure 12A**). The specificity and accessibility of PLA2-pep-3 were verified using one of the mAbs-n (PLA2-mc8, its amino acid sequence of the variable region is shown in **Supplementary Table 1**). Results showed that mAb-n PLA2-mc8 had the same specificity with the pAbs-p against PLA2-pep-3 (**Figure 5A**). Similarly, mAb-n (SVMP-mc18, its amino acid sequence of the variable region is shown in **Supplementary Table 1**) against SVMP showed the same specificity as pAbs-p against SVMP-pep-1 (**Figure 5C** and **Supplementary Figure 12B**). The pAbs-n against PLA_2_ and SVMP (called pAbs-n-PLA2 and pAbs-n-SVMP, respectively) were purified using protein A-agarose gel, the concentration was 1 mg/ml, and their titers and specificities were analyzed. The purity of the purified pAbs-n was checked by SDS-PAGE (**Supplementary Figure 13**), and the titers of the pAbs-n-PLA2 and pAbs-n-SVMP were both higher than 1:128,000 (**Figures 5B, D** **)**. The ELISA and Western blotting results showed that the pAbs-n-PLA2 had good specificity (**Figure 5B**), while the pAbs-n-SVMP had lower specificity and obvious cross-reaction with *T. stejnegeri*, *D. r. siamensis*, and *D. acutus* (**Figure 5D**).

### Establishment of ELISA and Venom Testing

**Figure 6:**
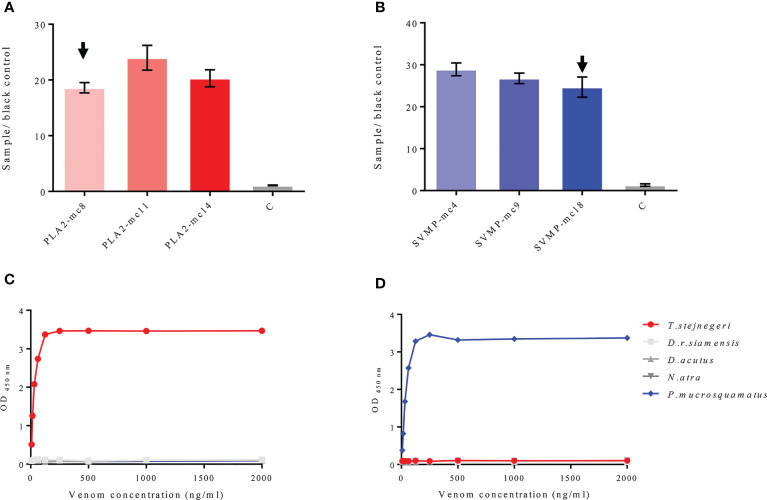
Matching of mAbs-n and pAbs-n and testing on natural antigens. mAbs-n against PLA2-pep-3 and PLA_2_ protein and mAbs-n against SVMP-pep-1 and SVMP protein were matched with anti-PLA_2_ pAbs-n and anti-SVMP pAbs-n, respectively. pAbs-n with HRP-labeled mAbs-n as the detector antibody showed high OD_450 nm_ and low detection limit (7.81 ng/ml). Antibody matching of PLA2-mc8, PLA2-mc11, and PLA2-mc14 (positive mAbs-n to PLA2-pep-3 and PLA_2_) with pAbs-n was successful **(A)**, with PLA2-mc8 showing the best matching effect **(C)**. In the antibody pairing test, SVMP-mc4, SVMP-mc9, and SVMP-mc18 (positive mAbs-n to SVMP-pep-1 and SVMP) matched well with pAbs-n **(B)**, with SVMP-mc18 showing the best matching effect **(D)**.

Positive mAbs-n to PLA2-pep-3 and PLA_2_ protein and to SVMP-pep-1 and SVMP protein were matched with pAbs-n-PLA2 and pAbs-n-SVMP, respectively. Antibody matching was carried out as follows. The pAbs-n were coated with HRP-labeled mAbs-n as the detecting antibody and vice versa. ELISA showed that pAbs-n with HRP-labeled mAbs-n were the best, i.e., high OD_450 nm_ and low detection limit (7.81 ng/ml) (**Supplementary Figures 14, 15**). In the antibody matching tests for PLA2-mc8, PLA2-mc11, and PLA2-mc14, pAbs-n-PLA2 matching was successful (**Figure 6A**), and PLA2-mc8 showed the lowest detection limit (**Figure 6C**). Similarly, pAbs-n-SVMP matching was also successful with SVMP-mc4, SVMP-mc9, and SVMP-mc18 (**Figure 6B**), and SVMP-mc18 had a good matching effect and low detection limit (**Figure 6D**). So, pAbs-n-PLA2 and PLA2-mc8 and pAbs-n-SVMP and SVMP-mc18 were used to antibody pairing to establish the ELISA method and to test the venom of *T. stejnegeri* and *P. mucrosquamatus*, respectively.

**Figure 7:**
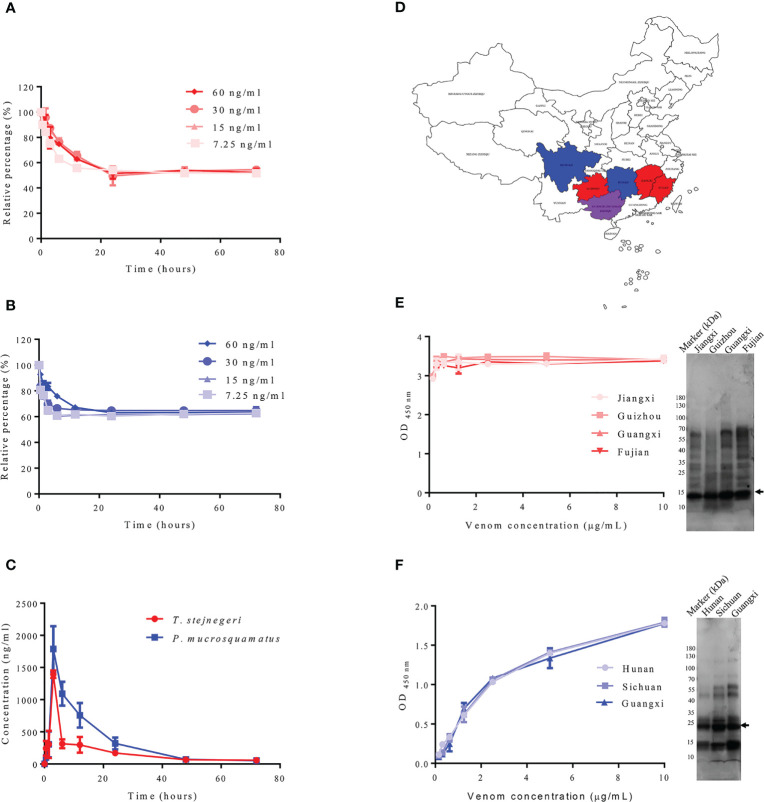
Detection of samples containing snake venom by sandwich ELISA. Double-antibody sandwich ELISA was used to detect snake venom in plasma samples. Using *in vitro* experiments, different concentrations of *T. stejnegeri* and *P. mucrosquamatus* venom were incubated in plasma from healthy human donors for different times at 37°C. Results showed that *T. stejnegeri* **(A)** and *P. mucrosquamatus* venom **(B)** in plasma decreased in the first 10 h and then remained at a relatively constant concentration. For *in vivo* simulation experiments, venom content in plasma increased gradually and peaked at 3 h, then decreased until it was undetectable after 48 h **(C)**. Venom of *T. stejnegeri* and *P. mucrosquamatus* from different habitats was detected by double-antibody sandwich ELISA. Venom of *T. stejnegeri* snakes from Jiangxi, Guizhou, Guangxi, and Fujian provinces in China [**(D)**, red and purple] and venom of *P. mucrosquamatus* snakes from Hunan, Sichuan, and Guangxi provinces in China [**(D)**, blue and purple] were collected and compared by ELISA and SDS-PAGE. Some differences were detected in the snake venom content from various areas [**(E**, **F)**, right], but contents of PLA_2_ [**(E)**, left] and SVMP varied a little [**(F)**, left]. PLA_2_ and SVMP are indicated by arrows.

Although the composition of venom is influenced by various factors, including climatic zone, diet, ontogenetic shifts, life stage, and predation pressure (18), highly abundant toxins are less affected (19). To determine whether PLA_2_ and SVMP are suitable for the diagnosis of snakebite from different habitats, the contents of PLA_2_ and SVMP in *T. stejnegeri* and *P. mucrosquamatus* from different habitats were compared. Results showed some differences in the components of snake venom from different habitats, but the content of PLA_2_ and SVMP varied a little (**Figures 7D–F**).

The detection capacity of snake venom using the prepared ELISA kit for detecting PLA_2_ and SVMP was verified *in vitro* and *in vivo*, and the venom concentrations were determined according to the established calibration curves (**Supplementary Figure 16**), with a detection limit of 6.25 ng/ml. According to literature reports, most snake venoms can bind to serum proteins (20–22). In order to prove whether PLA_2_ and SVMP also bind to plasma proteins, and whether it is affected by plasma protein when determining its concentration, different concentrations of snake venom from *T. stejnegeri* and *P. mucrosquamatus* were added to the plasma from healthy donors and incubated at 37°C at different times. Plasma samples (100 μl) were removed at different time points and frozen at −20°C. After the sampling process, the ELISA kit was used to detect the content of the corresponding snake venom proteins in plasma. Results showed that the detectable *T. stejnegeri* (**Figure 7A**) and *P. mucrosquamatus* (**Figure 7B**) venom proteins in plasma decreased at first and then remained at a relatively constant concentration. In the simulated snakebite envenomation experiment, rats were injected with 1 mg of *T. stejnegeri* or *P. mucrosquamatus* venom in the right hind leg muscle, and 40–50 μl of blood was taken from the tail vein at different time points. After 1 h at room temperature, the blood was centrifuged at 3,000×*g* for 20 min at 4°C, and the resulting plasma was diluted 50 times with PBS. After the sampling process, the content of snake venom in the plasma was detected by the ELISA kit. Results showed that snake venom content in the plasma increased gradually and peaked at 3 h, then decreased until it was undetectable after 48 h (**Figure 7C**).

## Discussion

Despite many challenges, the World Health Organization (WHO) announced its intention to take action on a globally coordinated response strategy for snakebites (23). Successful snakebite treatment requires the development of cross-regional, comprehensive, effective, affordable, and stable diagnosis tools and medicines. The major issue with snakebite diagnosis is the cross-reactivity among venoms, as some proteins in each venom overlap and detection devices are rarely species-specific, instead detecting a range of species when using immunological techniques (24). To date, most diagnostic antibodies exhibit poor specificity (12–15), thus hindering the correct selection and dosage of antiserum. We showed here that the principles of venom similarities and the underlying cross-reactivities of the same genus could be applied to develop universal genus-specific diagnosis tools for snakebite (11). A strategy was developed to effectively choose and prepare genus-specific snake venom antibodies that meet the requirement of a rapid and efficient clinical diagnosis of snakebite. Based on the strategy, double-antibody sandwich ELISA was established using antibodies that can accurately and efficiently identify venoms from snake species belonging to the genera of *Trimeresurus* and *Protobothrops*, which are a sister clade of pit vipers. The current method showed a detection limit of 6.25 ng/ml on the snake venoms and a little cross-reaction with other genus snake venoms, thus proposing an effective and affordable diagnosis strategy with high feasibility and applicability. According to the report of Liu et al., the average venom concentrations in plasma are 8.93 ng/ml (*n* = 4) and 29.48 ng/ml (*n* = 6) produced by *T. stejnegeri* and *P. mucrosquamatus* bite, respectively (12). So, the achieved detection limit of the proposed method is compatible with real-life diagnosis situations.

Following envenomation, the lymphatic system absorbs high-molecular weight toxins, whereas the blood vascular endothelium absorbs low-molecular weight toxins (25). At the same time, venom components, particularly the non-enzymatic toxins, bind very tightly to circulatory proteins and/or their target tissues, and a small quantity of venom may be available in circulation and/or body fluids for detection. Thus, low-molecular weight and high-content enzymatic toxins are ideal antigens for the preparation of diagnostic antibodies. Thus, phospholipase A_2_ and snake venom metalloproteinase TM-3 are ideal proteins for the identification of *Trimeresurus* and *Protobothrops* snakes, respectively (**Figure 2**).

Proteomics data mining and analysis of polypeptide antigen specificity and accessibility to obtain genus-specific peptide antigens will improve the development of diagnostic antibodies. Considerable bioinformatics analysis has been carried out in the early stages of this study, including genus-specific (**Supplementary Figures 1, 2**), antigen immunogenicity (**Supplementary Figures 3, 4**), and three-dimensional structure analyses (**Supplementary Figures 5, 6**). Antigen immunogenicity prediction parameters include hydrophilicity, surface accessibility, antigenicity, flexibility, and secondary structure prediction. As a linear epitope of B cell, it should first be located on the protein surface, which is conducive to binding with an antibody. In addition, it should also have certain flexibility, which is helpful to the change of protein conformation when the antigen binds with the antibody (26). The immunogenicity of epitopes is determined by the physicochemical property of an amino acid and is little affected by individual amino substitution with similar properties (**Supplementary Figure 17**) (27). In this study, the epitopes of PLA2-pep-3 and SVMP-pep-1 are unique to *Trimeresurus* and *Protobothrops*, respectively, and PLA2-pep-3 has some amino substitution with similar properties, e.g., I→L and L→T (**Figures 2D, E** **)**.

Toxins from the same genus living in a similar environment are highly consistent but are quite different among different genera (28–30). Accurate diagnosis at the genus level will reduce the number of potential diagnostic targets from hundreds to dozens. In this study, the anti-PLA_2_ antibody successively identified 2 of the 32 species of *Trimeresurus*, due to extensive sequence similarities, and it is very likely that the developed anti-PLA_2_ antibodies will also be able to identify the venom of other *Trimeresurus* species. The anti-SVMP antibody identified 4 of the 12 species of *Protobothrops* by protein homology analysis (**Supplementary Figures 1, 2, 17**) (1).

Although considerable bioinformatics analysis has been carried out in the early stages of this study, experimental results showed that the specificity, immunogenicity, and surface accessibility of the peptides were not always consistent with the bioinformatics results. PLA2-pep-1, PLA2-pep-3, and SVMP-pep-1 were consistent with the bioinformatics results, whereas SVMP-pep-2 and SVMP-pep-3 were quite different. Analysis indicated that SVMP-pep-2 and SVMP-pep-3 were genus-specific, surface accessible, and strongly immunogenic peptides, while the experimental results showed that SVMP-pep-2 was surface accessible but not specific and SVMP-pep-3 had poor immunogenicity. It is an unexplained phenomenon in this study that pAbs-p perform well in Western blot assays, recognizing specifically their correlate venoms, but perform poorly in ELISA assays. So far, we cannot give a more reasonable explanation and there is no corresponding literature to explain this phenomenon. Here, we speculate that it may be caused by the sensitivity of WB and ELISA. That is, ELISA has a higher sensitivity than WB (**Figure 3**).

Here, for the development of diagnostic antibodies for *Trimeresurus* and *Protobothrops*, only 30 days was required to prepare the anti-polypeptide pAbs, while 180 days was required to prepare the mAbs, and the cost of preparing the pAbs was significantly lower than that of mAbs (US$800 and US$5,000, respectively). Therefore, the preparation of polyclonal antibodies against the selected peptides and validation of specificity, immunogenicity, and surface accessibility of the peptides by these pAbs in advance have become essential steps, and the time and cost of development can be greatly reduced.

Most commercially available immunological diagnostic reagents usually contain mAbs against specific epitopes of a single antigen, and the methods used to capture and coat antibodies in the same diagnostic kit are different. However, it is very difficult to match small-molecular weight proteins with two mAbs against a linear epitope. In order to solve this problem and improve the success rate of antibody pairing, high affinity polyclonal antibodies against natural antigens can be prepared by natural antigens. As natural antigens have multiple antigenic epitopes (including linear and spatial epitopes), the possibility of successful matching is increased. The synthetic short peptide antigen is a low-molecular weight antigen and has a different spatial structure to the natural protein antigen. Therefore, antibodies prepared by these peptides exhibit weak binding affinity to the natural antigen (**Supplementary Figure 9**) and vice versa (**Supplementary Figure 10**). Therefore, mAbs-n and pAbs-n against the natural protein antigen were used in this study.

To verify the universality of this antibody development strategy, we analyze the venom of snakes of Viperidae in North America in the same way, which include the genera *Crotalus*, *Sistrurus*, and *Agkistrodon* and cause most of the snakebite envenoming in North America (31). The analysis result showed that three GSAEPs, namely, IPSYDNKYWLF, KYWRFPTENCQ, and YDSKTYWKYPKK derived from the PLA_2_ of *C. atrox*, *S. miliarius*, and *A. piscivorus*, respectively, which are representing species of the genera *Crotalus*, *Sistrurus*, and *Agkistrodon* correspondingly, were found to identify the genera *Crotalus* (**Supplementary Figure 18A**), *Sistrurus* (**Supplementary Figure 18B**), and *Agkistrodon* (**Supplementary Figure 18C**), respectively. The two GSAEPs (IPSYDNKYWLF and KYWRFPTENCQ) can cover 8 of the total 12 species of rattlesnakes (32), and the YDSKTYWKYPKK can identify copperheads (*A. c. contortrix*) and cottonmouths (*A. piscivorus*). The 3D structure of the PLA_2_s obtained through homology modeling and the protein structure alignment results showed that the three GSAEPs were located in a similar spatial position as PLA2-pep-3 of *T. stejnegeri*, and GSAEPs were highlighted by a black box (**Supplementary Figures 18D, E**). Antigenicity analysis results of the three GSAEPs in **Supplementary Figure 19** showed that they were hydrophilic, immunogenic, and surface accessible.

With the continuous accumulation of bioinformatics data and the deepening of research, we believe that the proposed strategy will help produce specific diagnostic antibodies and clinical diagnostic reagents and, thus, provide important guidance for accurate snakebite treatment.

## Materials and Methods

### Snake Venom, Peptides, and Animals

Venom samples from wild *T. stejnegeri*, *D. r. siamensis*, *D. acutus*, *N. atra*, and *P. mucrosquamatus* snakes were collected in China according to previous study protocols (33, 34). The samples were centrifuged at 12,000×*g* for 10 min at 4°C to remove debris, then lyophilized, and stored at −80°C until use. All peptides in this paper were biochemically synthesized by GL Biochem (Shanghai, China).

All experiments involving snakes, rabbits, rats, and mice were approved by the Animal Care and Use Committee at the Kunming Institute of Zoology, Chinese Academy of Sciences, Yunnan, China (SMKX-20190729-108).

### Bioinformatics Analysis

Based on the existing transcriptomics and proteomics data, specific antigenic proteins with high content were selected from venomous snake species of interest. Several factors are considered when selecting peptide antigens, including sequence homology, secondary structure, hydrophilicity, immunogenicity, and surface accessibility. Here, the main criteria for peptide antigen selection included the following: 1) homologous sequences with 75%–100% consistency and 90%–100% sequence coverage for PLA_2_ and homologous sequences with 60%–100% consistency and 90%–100% sequence coverage for SVMP (BLAST at NCBI); 2) peptides must be located on the surface of the structural models (SWISS-MODEL); and 3) using the Protean module in Lasergene, the secondary structure of the peptides must contain no or few α-turns and β-sheets based on Chou–Fasman analysis; the average hydrophilicity of the peptides must be >2.0 based on Kyte–Doolittle analysis; the average antigenic index of the peptides must be >1.5 based on Jameson–Wolf analysis; and the average surface probability must be >1.0 based on Emini analysis. The information of selected antigens is summarized in **Figure 2**.

### Purification of PLA_2_ and SVMP

PLA_2_ and SVMP were purified from the crude venom of *T. stejnegeri* and *P. mucrosquamatus*, respectively, by fast protein liquid chromatography (FPLC) (AKTA pure, GE Healthcare, Boston, MA, USA) with a cation exchange column (Resource™ S, 6 ml, GE Healthcare, Boston, MA, USA), with elution flow rates of 2 and 3 ml/min, respectively. In brief, 50 mg of crude *T. stejnegeri* or *P. mucrosquamatus* venom was dissolved in 1 ml of buffer A (8 mM NaHPO_4_·12H_2_O, 1.5 mM KH_2_PO_4_, pH 7.4), then filtered through 0.22-μm sterile filters (SLGPR33RB, Millipore, Burlington, MA, USA), and loaded into the cation exchange column pre-equilibrated in buffer A. Subsequently, the column was washed with buffer A to elute non-binding proteins, and binding proteins were eluted by applying a salt gradient from 0 to 1 M NaCl in buffer A. Finally, the column was equilibrated with buffer A to prepare for the next sample load. During the elution process, each fraction was collected according to the absorbance peaks at 215 nm and lyophilized for further analysis.

### Preparation of the KLH–Peptide Complex Antigen

The peptides were biochemically synthesized and conjugated with keyhole limpet hemocyanin (KLH) to prepare the KLH–peptide complex antigens. These antigens were prepared using a Maleimide-Activated KLH–Peptide Conjugation Kit (K2039-5, BioVision, Milpitas, CA, USA) according to the instructions of the manufacturer.

### Immunization of Rabbits and Collection of Plasma Samples

The KLH–peptide complex antigens were dissolved in saline at 2 mg/ml and filtered through 0.22-μm sterile filters. The antigens (0.5 ml) were emulsified with an equal volume of Freund’s complete adjuvant (F-5881, Sigma, Darmstadt, Germany) and injected intradermally at multiple sites along the backs of male New Zealand white rabbits. Two rabbits were injected with one kind of antigen. Booster doses were made in Freund’s incomplete adjuvant (F-5506, Sigma, Darmstadt, Germany) at 2-week intervals with 500-μg doses of the same antigen, totally two times with intervals of 2 weeks. Rabbit serum was collected 14 days after the second booster injection. Antibody titers were detected by indirect ELISA using the respective antigen as coating antigen and the pre-immunization serum of each rabbit as the negative control.

### Generation and Sequences of mAbs-n

The anti-PLA_2_ and anti-SVMP mAbs-n were prepared according to a previous study (35) with slight modification. Five female BALB/c mice (6–8 weeks old) were immunized with PLA_2_ or SVMP. Each mouse was immunized four times with 3-week intervals. For the first immunization, 100 μg of PLA_2_ or SVMP was emulsified in Freund’s complete adjuvant (CFA) (F-5881, Sigma, Darmstadt, Germany). Subsequent immunizations (boosters) were administered three times successively with 100 μg of antigen in Freund’s incomplete adjuvant (IFA) (F-5506, Sigma, Darmstadt, Germany) with 3-week intervals. One week after the second and third boosters, blood samples were collected by tail bleeding to determine antiserum titers using indirect ELISA. The mice with higher antiserum titers were given an additional booster immunization at 7 days before cell fusion. Subsequently, hybridoma cell cloning and mAb production were carried out according to references.

The validated immunoglobulin variable chain (IgV) sequences were sequenced by GENEWIZ (Jiangsu, China) as per prior study (36).

### Antibody Purification

Serum from hyperimmunized rabbits with higher antibody titers was subjected to purification. IgG was purified by affinity chromatography on a FPLC system with a 5-ml HiTrap Protein A column (45-002-379, GE Healthcare, Boston, MA, USA) according to the instructions of the manufacturer. Briefly, the column was washed with 10 column volumes of binding buffer [phosphate-buffered saline (PBS), containing 137 mM NaCl, 2.7 mM KCl, 8 mM NaHPO_4_·12H_2_O, 1.5 mM KH_2_PO_4_, pH 7.4] until no material appeared in the effluent. Binding IgG was eluted with five-column volumes of elution buffer (0.1 M sodium citrate, pH 3.6). The eluted IgG solution was neutralized to pH 7.0 by adding a neutralization solution (1 M Tris–HCl, pH 9.0) and was concentrated and desalinated by ultrafiltration with 50-kDa hyperfiltration tubes (UFC805024, Millipore, Burlington, MA, USA). The antibody concentrations were determined using a BCA kit (T9300A, TaKaRa, Dalian, China), and the antibodies were then stored at −80°C.

### Affinity Purification of pAbs-p

Antibodies in the serum of rabbits hyperimmunized with the KLH–peptide complex antigen were purified by affinity chromatography as per previous study (12). Briefly, CNBr-activated Sepharose 4B beads (17043001, GE Healthcare, Boston, MA, USA) were coupled with each peptide (10 mg) for use as the affinity medium and packed into a 5-ml column to capture specific antibodies.

To purify the anti-PLA2-pep-1, anti-PLA2-pep-3, anti-SVMP-pep-1, anti-SVMP-pep-2, and anti-SVMP-pep-3 antibodies, 2 ml of serum from each rabbit was diluted in 30 ml of binding buffer and then pumped into the corresponding affinity column at 4°C for 3 h (repeated twice). The affinity columns were then washed with binding buffer and washing buffer. After washing, each column was eluted with elution buffer, and the eluted fractions were collected into microcentrifuge tubes containing neutralized buffer. Finally, all eluted fractions were concentrated and desalinated by ultrafiltration with 50-kDa hyperfiltration tubes (UFC805024, Millipore, Burlington, MA, USA). The concentration of antibodies was determined using a BCA kit (T9300A, TaKaRa, Dalian, China), and the antibodies were then stored at −80°C.

### Indirect ELISA

Each well of a 96-well ELISA microplate (2506, Corning, Kennebunk, ME, USA) was coated with 100 μl (the concentration of antigen has been marked in the figure legend) of the corresponding antigen in PBS overnight at 4°C. After being washed three times with PBST (PBS, containing 0.05% Tween-20), the plate was blocked with 250 μl of blocking solution [3% bovine serum albumin (BSA) in PBST] at 37°C for 1 h. After the plate was washed three times with PBST, 100 μl of diluted antiserum or antibody in dilution buffer (3% BSA in PBST) was added into each well, followed by incubation at 37°C for 1 h. The plate was washed three times with PBST and then incubated with HRP-labeled anti-rabbit IgG antibody (7074s, CST, Danvers, MA, USA) or anti-mouse IgG antibody (7076s, CST, Danvers, MA, USA) diluted 1:3,000 with PBST at 37°C for 1 h. The plate was washed three times with PBST to remove unbound secondary antibodies. Finally, 100 μl of peroxide substrate solution (PR1200, Solarbio, Beijing, China) was added to each well, followed by incubation at room temperature for 10–20 min. Color development was ended by adding 100 μl of 2.5 M sulfuric acid (C1058, Solarbio, Beijing, China) per well, and absorbance was recorded at 450 nm with a microplate reader (Epoch, Bio-Tech, Minneapolis, MN, USA).

### Western Blot Analysis

Samples were separated by 12% reduced SDS-PAGE using Mini-PROTEAN (Bio-Rad, Hercules, CA, USA). After electrophoresis, the proteins on the gel were either stained with Coomassie Brilliant Blue R250 (ST031, Beyotime, Shanghai, China) or transferred to a 0.45-μm polyvinylidene fluoride (PVDF) membrane (IPVH00010, Millipore, Burlington, MA, USA). The membrane was then blocked with 5% BSA (ABIO-Biofroxx-0037E, Biofroxx, Germany) in washing buffer (PBST, pH 7.4) for 1 h at room temperature on a horizontal shaker (Qilinbeier, Jiangsu, China). After being washed with PBST once, the membrane was incubated with a suitably diluted antiserum (all of the pAbs-ps were diluted 3,000 times, except pAbs-p against SVMP-pep-3 which was diluted 1,000 times)/mAbs-n (all of the mAbs-ns were diluted 3,000 times) overnight at 4°C. After being washed with PBST three times, the proteins on the PVDF membranes were detected by incubation with appropriate HRP-conjugated anti-rabbit IgG antibody (diluted 3,000 times) or anti-mouse IgG antibody (diluted 3,000 times) for 1 h at room temperature, and then the PVDF membranes were washed with PBST. The PVDF membranes were imaged with a chemiluminescence imaging system (5200 Multi, Tanon, Shanghai, China), with the ECL solution (180-501, Tanon, Shanghai, China) used as the luminescent substrate.

### Preparation of HRP-Labeled Antibodies

The HRP-labeled antibodies were prepared using an HRP Conjugation Kit (ab102890, Abcam, Cambridge, UK) according to the instructions of the manufacturer. Briefly, before adding the antibody to the HRP mix, 1 µl of the modifier reagent was added to 10 µl of each antibody to be labeled and mixed gently. Each antibody sample (with added modifier reagent) was pipetted directly onto the lyophilized material of the HRP conjugation mix and resuspended gently by withdrawing and redispensing the liquid once or twice using a pipette. After capping the vial, the mixed solution was left standing in the dark at room temperature (20°C–25°C) for 3 h. Following incubation for 3 h (or more), 1 µl of the quencher reagent was added to 10 µl of each antibody and mixed gently. The conjugate could be used after 30 min and did not require purification.

### Development of Sandwich ELISA

The pAbs-n (100 μl at 10 μg/ml) in PBS were coated in each well of a 96-well ELISA microplate (2506, Corning, Kennebunk, ME, USA) for 1 h at 37°C. Thereafter, the wells were blocked by incubation with 3% BSA in PBS for 1 h and washed three times using 250 μl of PBST per well. The samples (100 μl) to be tested were added to individual wells, followed by incubation at 37°C for 1 h. After washing three times with PBST, 100 μl of the HRP-labeled mAbs-n (diluted 1:3,000 in PBST) was added to each well and the plate was incubated for 1 h at 37°C. Subsequently, the plate was washed three times with PBST, and 100 μl of peroxide substrate solution (PR1200, Solarbio, Beijing, China) was added to each well, followed by incubation at room temperature for 10–20 min. Color development was ended by adding 100 μl of 2.5 M sulfuric acid (C1058, Solarbio, Beijing, China) to each well, and absorbance was recorded at 450 nm with a microplate reader (Epoch, Bio-Tech, Minneapolis, MN, USA).

### Simulation Experiment of Snakebite Envenomation *In Vivo* and *In Vitro*

Simulation experiments were performed in male rats (SD strain, body weight 200 g). The rats were maintained under specific pathogen-free conditions at a temperature of 22°C and humidity level of 60%–70% and were provided with food and water *ad libitum*. Rats (*n *= 3/group) were injected in the hind leg muscle with 1 mg of venom dissolved in sterile saline. Blood samples from each rat were collected using a heparinized capillary blood collection system (KMIC-EDTA, Kent Scientific, Torrington, CT, USA) at 0.5, 1.5, 3, 6, 12, 24, 48, and 72 h after venom injection. The blood samples were centrifuged at 3,000×*g* for 20 min at 4°C after standing at room temperature for 30 min. The supernatant (plasma) was collected into a microcentrifuge tube and stored at −80°C.

Different concentrations of *T. stejnegeri* and *P. mucrosquamatus* venom were added to the plasma from healthy donors and incubated at 37°C. Plasma samples (100 μl) were taken at different time points (0.5, 1.5, 3, 6, 12, 24, 48, and 72 h) and frozen at −20°C. After the sampling process, double-antibody sandwich ELISA was used to detect the content of the corresponding snake venom proteins in the plasma.

### Statistical Analysis

All data were plotted and analyzed using GraphPad Prism 6 software (GraphPad Software Inc., San Diego, CA, USA) and shown as means ± standard error of the mean (SEM). Statistical significance was determined by unpaired Student’s *t*-test. *P*-values <0.05 were considered statistically significant (*P < 0.05; **P < 0.001).

## Data Availability Statement

The original contributions presented in the study are included in the article/**Supplementary Material**. Further inquiries can be directed to the corresponding author.

## Ethics Statement

The animal study was reviewed and approved by the Animal Care and Use Committee at the Kunming Institute of Zoology, Chinese Academy of Sciences, Yunnan, China (SMKX-20190729-108). Written informed consent was obtained from the owners for the participation of their animals in this study.

## Author Contributions

Conception and design: CL, QL, and RL. Development of the methodology: CL, FW, QL, and RL. Acquisition of data (provided animals, provided facilities, etc.): CL, FW, PL, YY, YD, and QL. Analysis and interpretation of data (e.g., statistical analysis): CL, BX, and JL. Writing, review, and/or revision of the manuscript: CL, QL, CS, and RL. Administrative, technical, or material support (i.e., reporting or organizing data, constructing databases): CL, BX, and RL. Study supervision: QL and RL. All authors contributed to the article and approved the submitted version.

## Funding

This work was supported by the National Science Foundation of China (31930015), Ministry of Science and Technology of China (2018YFA0801403), Chinese Academy of Sciences (XDB31000000, KGFZD-135-17-011, and SAJC202103), Science and Technology Department of Yunnan Province (202003AD150008, 2019ZF003, 202002AA100007, and 202001AT070116), and Science and Technology Department Guangxi Zhuang Autonomous Region (Z20200287).

## Conflict of Interest

The authors declare that the research was conducted in the absence of any commercial or financial relationships that could be construed as a potential conflict of interest.

## Publisher’s Note

All claims expressed in this article are solely those of the authors and do not necessarily represent those of their affiliated organizations, or those of the publisher, the editors and the reviewers. Any product that may be evaluated in this article, or claim that may be made by its manufacturer, is not guaranteed or endorsed by the publisher.

## References

[1] Alencar LRV Quental TB Grazziotin FG Alfaro ML Martins M Venzon M. Diversification in Vipers: Phylogenetic Relationships, Time of Divergence and Shifts in Speciation Rates. Mol Phylogenet Evol (2016) 105:50–62. doi: 10.1016/j.ympev.2016.07.029 27480810

[2] Slowinski JB Keogh JS. Phylogenetic Relationships of Elapid Snakes Based on Cytochrome B mtDNA Sequences. Mol Phylogenet Evol (2000) 15:157–64. doi: 10.1006/mpev.1999.0725 10764543

[3] Gutierrez JM Calvete JJ Habib AG Harrison RA Williams DJ Warrell DA. Snakebite Envenoming. Nat Rev Dis Primers (2017) 3:17079. doi: 10.1038/nrdp.2017.79 28905944

[4] Tasoulis T Isbister GKA. Review and Database of Snake Venom Proteomes. Toxins (2017) 9:290. doi: 10.3390/Toxins9090290 PMC5618223 28927001

[5] Lomonte B Tsai WC Urena-Diaz JM Sanz L Mora-Obando D Sanchez EE. Venomics of New World Pit Vipers: Genus-Wide Comparisons of Venom Proteomes Across Agkistrodon. J Proteomics (2014) 96:103–16. doi: 10.1016/j.jprot.2013.10.036 PMC4294458 24211403

[6] Pla D Sanz L Molina-Sanchez P Zorita V Madrigal M Flores-Diaz M. Snake Venomics of Lachesis Muta Rhombeata and Genus-Wide Antivenomics Assessment of the Paraspecific Immunoreactivity of Two Antivenoms Evidence the High Compositional and Immunological Conservation Across Lachesis. J Proteomics (2013) 89:112–23. doi: 10.1016/j.jprot.2013.05.028 23747394

[7] Ainsworth S Petras D Engmark M Sussmuth RD Whiteley G Albulescu LO. The Medical Threat of Mamba Envenoming in Sub-Saharan Africa Revealed by Genus-Wide Analysis of Venom Composition, Toxicity and Antivenomics Profiling of Available Antivenoms. J Proteomics (2018) 172:173–89. doi: 10.1016/j.jprot.2017.08.016 28843532

[8] Khow O Pakmanee N Chanhome L Sriprapat S Omori-Satoh T Sitprija V. Cross-Neutralization of Thai Cobra (Naja Kaouthia) and Spitting Cobra (Naja Siamensis) Venoms by Thai Cobra Antivenom. Toxicon (1997) 35:1649–51. doi: 10.1016/S0041-0101(97)00035-4 9428112

[9] Lin JH Sung WC Liao JW Hung DZA. Rapid and International Applicable Diagnostic Device for Cobra (Genus Naja) Snakebites. Toxins (Basel) (2020) 12:572. doi: 10.3390/toxins12090572 PMC7551368 32899472

[10] Hifumi T Sakai A Kondo Y Yamamoto A Morine N Ato M. Venomous Snake Bites: Clinical Diagnosis and Treatment. J Intensive Care (2015) 3:16. doi: 10.1186/s40560-015-0081-8 25866646 PMC4393627

[11] Knudsen C Jurgensen JA Fons S Haack AM Friis RUW Dam SH. Snakebite Envenoming Diagnosis and Diagnostics. Front Immunol (2021) 12:661457. doi: 10.3389/fimmu.2021.661457 33995385 PMC8113877

[12] Liu CC Yu JS Wang PJ Hsiao YC Liu CH Chen YC. Development of Sandwich ELISA and Lateral Flow Strip Assays for Diagnosing Clinically Significant Snakebite in Taiwan. PloS neglected Trop Dis (2018) 12:e0007014. doi: 10.1371/journal.pntd.0007014 PMC6292642 30507945

[13] Maduwage KP Gawarammana IB Gutierrez JM Kottege C Dayaratne R Premawardena NP. Enzyme Immunoassays for Detection and Quantification of Venoms of Sri Lankan Snakes: Application in the Clinical Setting. PloS neglected Trop Dis (2020) 14:e0008668. doi: 10.1371/journal.pntd.0008668 PMC7561112 33017411

[14] Pukrittayakamee S Ratcliffe PJ Mcmichael AJ Warrell DA Bunnag DA. Competitive Radioimmunoassay Using a Monoclonal-Antibody to Detect the Factor-X Activator of Russell Viper Venom. Toxicon (1987) 25:721–9. doi: 10.1016/0041-0101(87)90122-X 3313812

[15] Amuy E Alape-Giron A Lomonte B Thelestam M Gutierrez JM. Development of Immunoassays for Determination of Circulating Venom Antigens During Envenomations by Coral Snakes (Micrurus Species). Toxicon (1997) 35:1605–16. doi: 10.1016/s0041-0101(97)00045-7 9428107

[16] Tsai IH Wang YM Chen YH Tsai TS Tu MC. Venom Phospholipases A2 of Bamboo Viper (Trimeresurus Stejnegeri): Molecular Characterization, Geographic Variations and Evidence of Multiple Ancestries. Biochem J (2004) 377:215–23. doi: 10.1042/BJ20030818 PMC1223832 12959640

[17] Villalta M Pla D Yang SL Sanz L Segura A Vargas M. Snake Venomics and Antivenomics of Protobothrops Mucrosquamatus and Viridovipera Stejnegeri From Taiwan: Keys to Understand the Variable Immune Response in Horses. J Proteomics (2012) 75:5628–45. doi: 10.1016/j.jprot.2012.08.008 22906718

[18] Alape-Giron A Sanz L Escolano J Flores-Diaz M Madrigal M Sasa M. Snake Venomics of the Lancehead Pitviper Bothrops Asper: Geographic, Individual, and Ontogenetic Variations. J Proteome Res (2008) 7:3556–71. doi: 10.1021/pr800332p 18557640

[19] Hatakeyama DM Tasima LJ Galizio ND Serino-Silva C Rodrigues CFB Stuginski DR. From Birth to Adulthood: An Analysis of the Brazilian Lancehead (Bothrops Moojeni) Venom at Different Life Stages. PloS One (2021) 16(6). doi: 10.1371/journal.pone.0253050 PMC8191990 34111213

[20] Gibbs HL Sanz L Perez A Ochoa A Hassinger ATB Holding ML. The Molecular Basis of Venom Resistance in a Rattlesnake-Squirrel Predator-Prey System. Mol Ecol (2020) 29:2871–88. doi: 10.1111/mec.15529 32593182

[21] Drabeck DH Rucavado A Hingst-Zaher E Cruz YP Dean AM Jansa SA. Resistance of South American Opossums to vWF-Binding Venom C-Type Lectins. Toxicon (2020) 178:92–9. doi: 10.1016/j.toxicon.2020.02.024 PMC8522506 32135198

[22] Perez-Peinado C Defaus S Sans-Comerma L Valle J Andreu D. Decoding the Human Serum Interactome of Snake-Derived Antimicrobial Peptide Ctn[15-34]: Toward an Explanation for Unusually Long Half-Life. J Proteomics (2019) 204:103372. doi: 10.1016/j.jprot.2019.04.022 31051282

[23] Williams DJ Faiz MA Abela-Ridder B Ainsworth S Bulfone TC Nickerson AD. Strategy for a Globally Coordinated Response to a Priority Neglected Tropical Disease: Snakebite Envenoming. PloS Negl Trop Dis (2019) 13:e0007059. doi: 10.1371/journal.pntd.0007059 30789906 PMC6383867

[24] Williams HF Layfield HJ Vallance T Patel K Bicknell AB Trim SA. The Urgent Need to Develop Novel Strategies for the Diagnosis and Treatment of Snakebites. Toxins (2019) 11:363. doi: 10.3390/toxins11060363 PMC6628419 31226842

[25] Wang W Chen N Shen X Cunningham P Fauty S Michel K. Lymphatic Transport and Catabolism of Therapeutic Proteins After Subcutaneous Administration to Rats and Dogs. Drug Metab Disposition: Biol Fate Chemicals (2012) 40:952–62. doi: 10.1124/dmd.111.043604 22328584

[26] El-Manzalawy Y Dobbs D Honavar VG. In Silico Prediction of Linear B-Cell Epitopes on Proteins. Methods Mol Biol (2017) 1484:255–64. doi: 10.1007/978-1-4939-6406-2_17 PMC8109234 27787831

[27] Schein CH Bowen DM Lewis JA Choi K Paul A van der Heden van Noort GJ. Physicochemical Property Consensus Sequences for Functional Analysis, Design of Multivalent Antigens and Targeted Antivirals. BMC Bioinf (2012) 13(Suppl 13):S9. doi: 10.1186/1471-2105-13-S13-S9 PMC3426803 23320474

[28] Roman-Dominguez L Neri-Castro E Vazquez Lopez H Garcia-Osorio B Archundia IG Ortiz-Medina JA. Biochemical and Immunochemical Characterization of Venoms From Snakes of the Genus Agkistrodon. Toxicon: X (2019) 4:100013. doi: 10.1016/j.toxcx.2019.100013 32550570 PMC7285990

[29] Senji Laxme RR Attarde S Khochare S Suranse V Martin G Casewell NR. Biogeographical Venom Variation in the Indian Spectacled Cobra (Naja Naja) Underscores the Pressing Need for Pan-India Efficacious Snakebite Therapy. PloS Negl Trop Dis (2021) 15:e0009150. doi: 10.1371/journal.pntd.0009150 33600405 PMC7924803

[30] Senji Laxme RR Khochare S Attarde S Suranse V Iyer A Casewell NR. Biogeographic Venom Variation in Russell's Viper (Daboia Russelii) and the Preclinical Inefficacy of Antivenom Therapy in Snakebite Hotspots. PloS Negl Trop Dis (2021) 15:e0009247. doi: 10.1371/journal.pntd.0009247 33764996 PMC7993602

[31] Corbett B Clark RF. North American Snake Envenomation. Emergency Med Clinics North America (2017) 35:339–54. doi: 10.1016/j.emc.2016.12.003 28411931

[32] Ruha AM Kleinschmidt KC Greene S Spyres MB Brent J Wax P. The Epidemiology, Clinical Course, and Management of Snakebites in the North American Snakebite Registry. J Med Toxicol Off J Am Coll Med Toxicol (2017) 13:309–20. doi: 10.1007/s13181-017-0633-5 PMC5711762 28975491

[33] Grego KF Vieira SEM Vidueiros JP Serapicos ED Barbarini CC da Silveira GPM. Maintenance of Venomous Snakes in Captivity for Venom Production at Butantan Institute From 1908 to the Present: A Scoping History. J Venom Anim Toxins (2021) 27:e20200068. doi: 10.1590/1678-9199-Jvatitd-2020-0068 PMC7856910 33597972

[34] Long C Liu M Tian H Li Y Wu F Mwangi J. Potential Role of Platelet-Activating C-Type Lectin-Like Proteins in Viper Envenomation Induced Thrombotic Microangiopathy Symptom. Toxins (2020) 12:749. doi: 10.3390/toxins12120749 PMC7760373 33260875

[35] Sepehr KS Baradaran B Majidi J Abdolalizadeh J Aghebati L Shahneh FZ. Development and Characterization of Monoclonal Antibodies Against Human CD20 in Balb/c Mice. Hum Antibodies (2012) 21:57–64. doi: 10.3233/HAB-130263 23549022

[36] Meyer L Lopez T Espinosa R Arias CF Vollmers C DuBois RM. A Simplified Workflow for Monoclonal Antibody Sequencing. PloS One (2019) 14:e0218717. doi: 10.1371/journal.pone.0218717 31233538 PMC6590890

