# Supplementary material for: A Strategy for Efficient Preparation of Genus-Specific Diagnostic Antibodies for Snakebites
Source: Front Immunol. 2021 Nov 9;12:775678. doi: 10.3389/fimmu.2021.775678 (PMC8660121; doi:10.3389/fimmu.2021.775678)
Supplement: Supplementary file 1 [file DataSheet_1.docx]

**Supplementary materials**

**
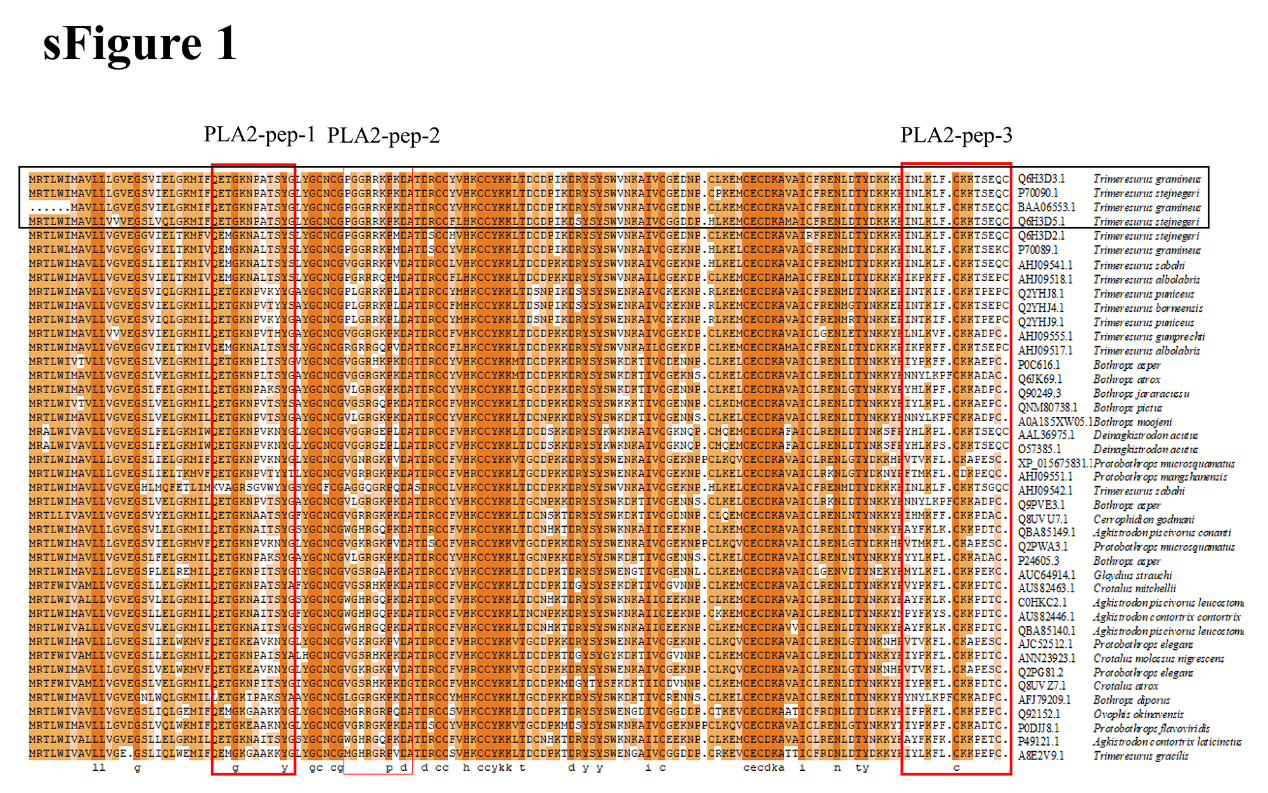
**

**Supplementary Figure 1 BLAST analysis of PLA_2_ protein sequence.** Based on BLAST analysis of NCBI database (https://blast.ncbi.nlm.nih.gov/Blast.cgi), homologous PLA_2_ sequences with 75%–100% consistency and 90%–100% sequence coverage were selected, then aligned using DNAMAN package. Sequence conservatism is represented by different shades of the same color, the color from dark to light corresponds to high to low sequence conservation. Three candidate PLA_2_ peptide antigens, i.e., QETGKNPATSYG (PLA2-pep-1), PGGRRKPK (PLA2-pep-2), and INLKLFCKKTSEQC (PLA2-pep-3), specific to *Trimeresurus* are shown in red box.


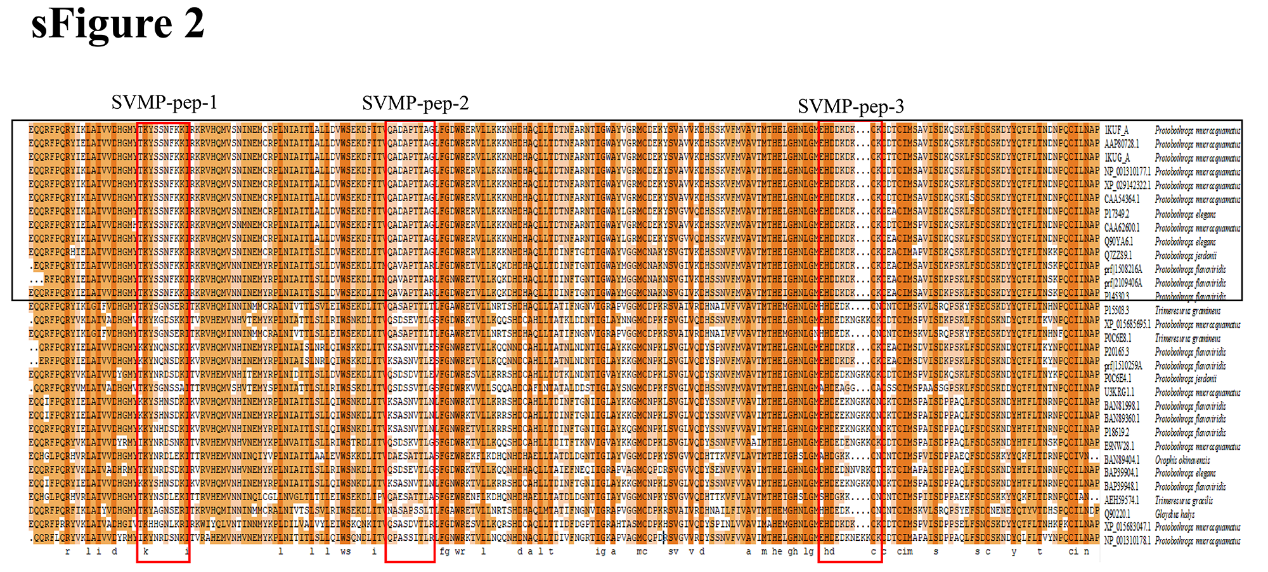


**Supplementary Figure 2 BLAST analysis of SVMP protein sequence.** Based on BLAST analysis of NCBI database (https://blast.ncbi.nlm.nih.gov/Blast.cgi), homologous SVMP sequences with 60%–100% consistency and 90%–100% sequence coverage were selected, then aligned using DNAMAN package. Sequence conservatism is represented by different shades of the same color, the color from dark to light corresponds to high to low sequence conservation. Three candidate SVMP peptide antigens, TKYSSNFKKI (SVMP-pep-1), QADAPTTAG (SVMP-pep-2), and EHDDKDKCK (SVMP-pep-3), specific to *Protobothrops* are shown in red box.

**
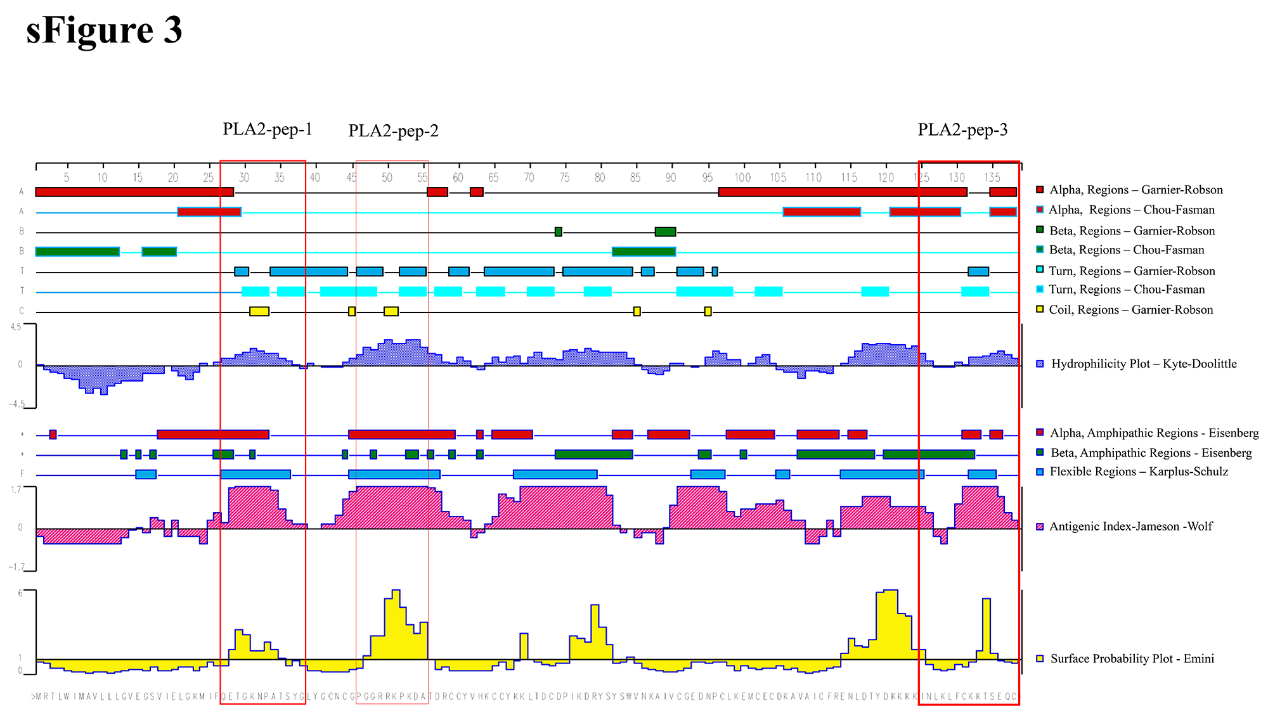
**

**Supplementary Figure 3 Antigenicity analysis of PLA_2_.** Protean module in Lasergene was used to analyze properties of PLA_2_ protein sequence, including secondary structure, hydrophilicity, immunogenicity, and spatial accessibility. Three peptides, i.e., PLA2-pep-1, PLA2-pep-2, and PLA2-pep-3 (shown in red box), were highly hydrophilic and immunogenic. PLA2-pep-1 and PLA2-pep-2 showed good surface accessibility, but PLA2-pep-3 showed relatively low surface accessibility.

**
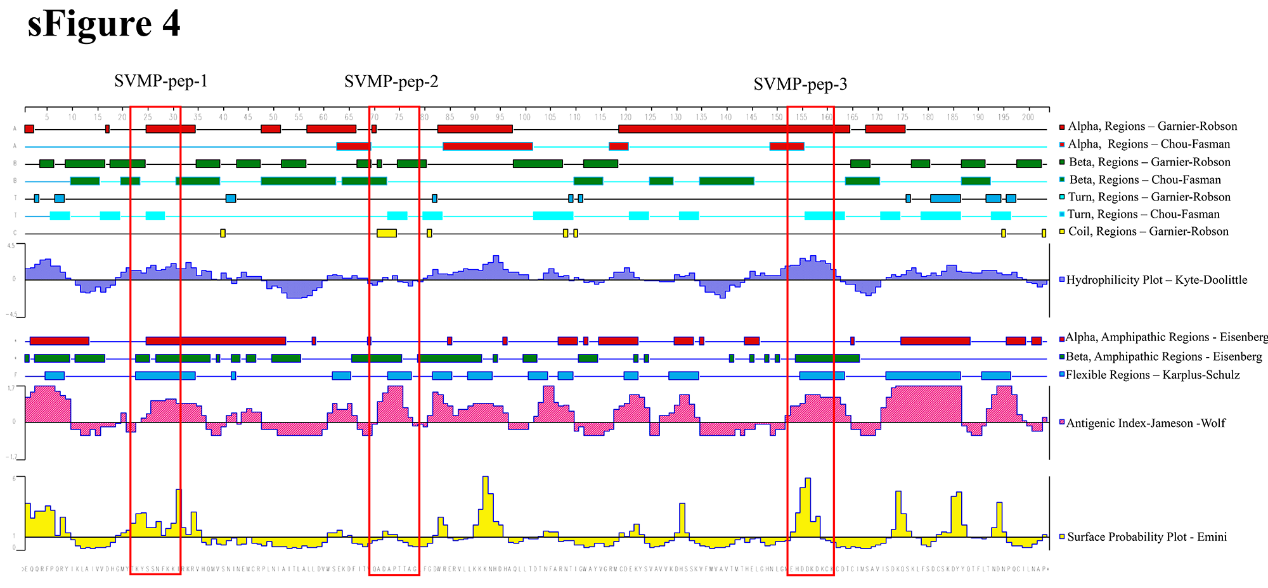
**

**Supplementary Figure 4 Antigenicity analysis of SVMP.** Protean module in Lasergene was used to analyze properties of SVMP protein sequence, including secondary structure, hydrophilicity, immunogenicity, and spatial accessibility. Among the three peptides (shown in red box), SVMP-pep-1 and SVMP-pep-3 were hydrophilic, immunogenic, and surface-accessible, while SVMP-pep-2 showed strong immunology but low hydrophilicity and surface accessibility.


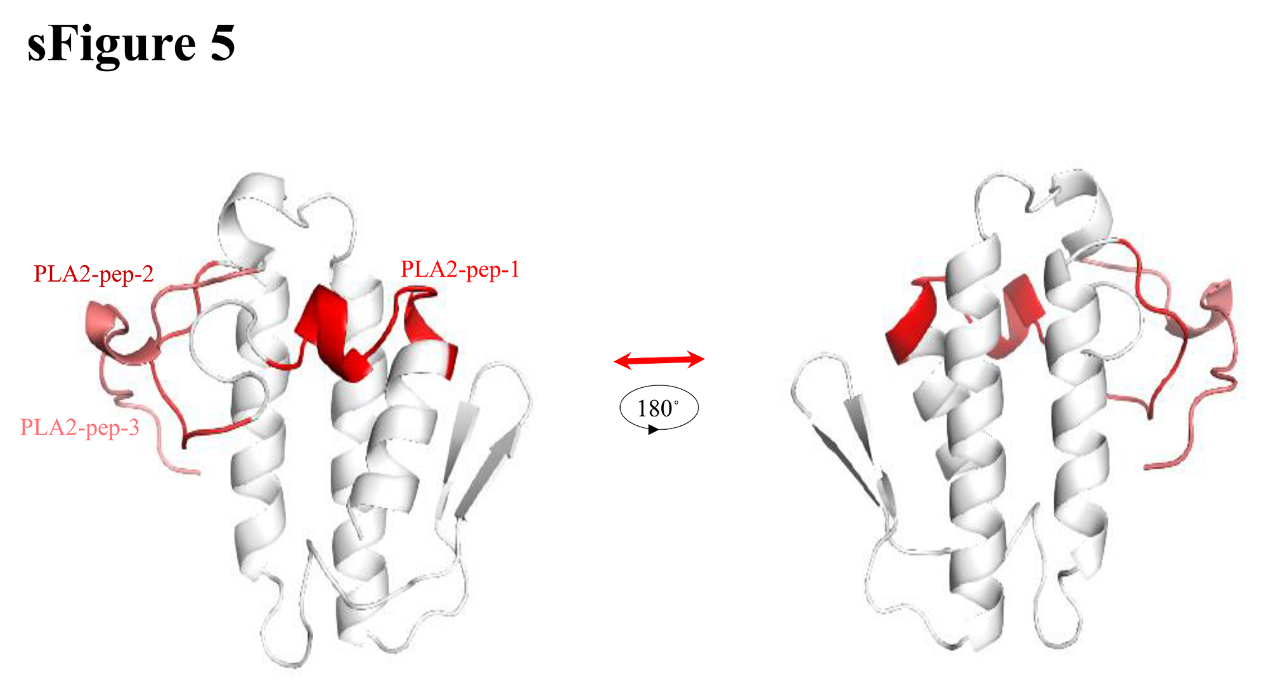


**Supplementary Figure 5 Structural homology modeling of PLA_2_.** Using SWISS-MODEL, structure of PLA_2_ from *Trimeresurus stejnegeri* was modeled using structure of PLA_2_ from *Deinagkistrodon acutus* (PDB No.: 1mc2.1. A) as a template. From the structure model, peptides PLA2-pep-1 and PLA2-pep-3 were located on protein surface, but peptide PLA2-pep-2 was shielded by peptide PLA2-pep-1. Therefore, peptide antigens of PLA2-pep-1 and PLA2-pep-3 were selected. To prepare complex antigen, a cysteine was added to the C-terminal of peptide PLA2-pep-1.

**
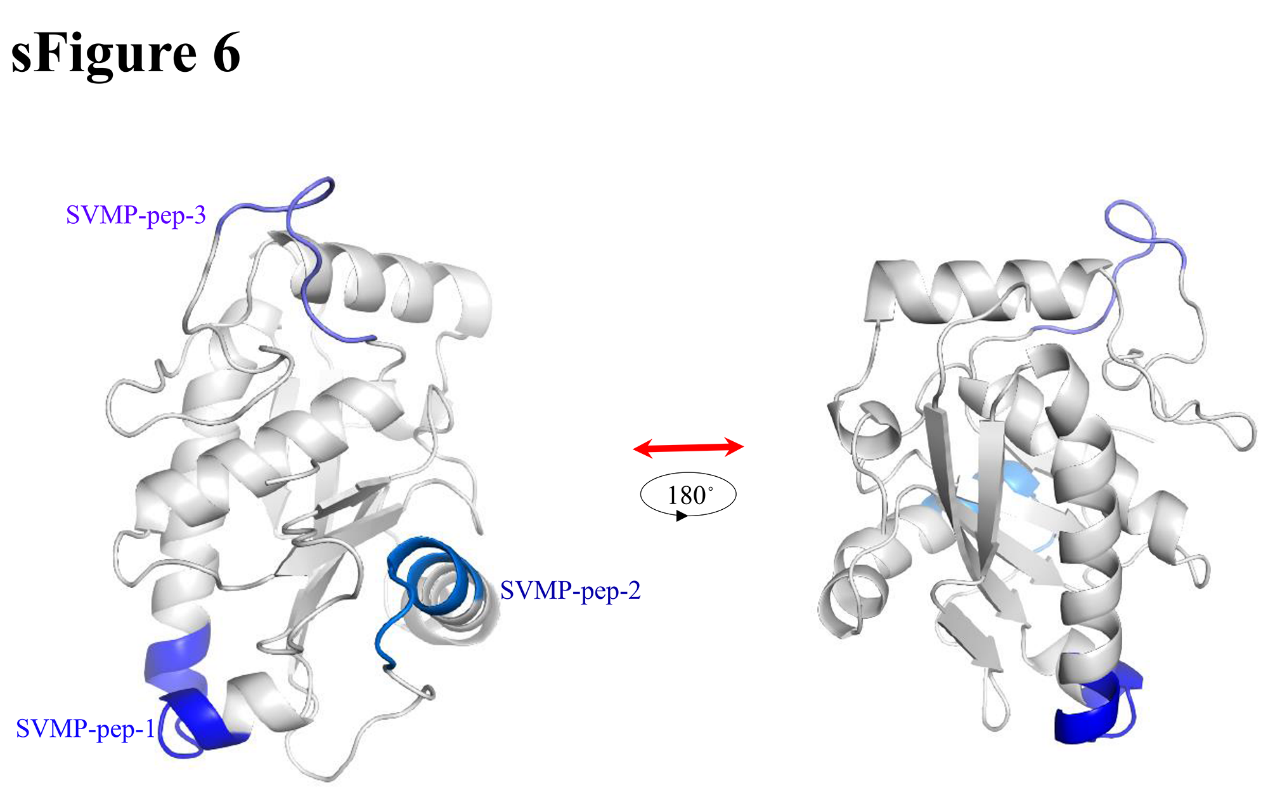
**

**Supplementary Figure 6 Structural homology modeling of SVMP.** Using SWISS-MODEL, structure of *Protobothrops mucrosquamatus* SVMP was obtained using structure of VAP2 from *Crotalus atrox* (PDB No.: 2dw2.1.A) as a template. From the structure, peptides SVMP-pep-1, SVMP-pep-1, and SVMP-pep-3 were located on protein surface. Therefore, three peptide antigens were selected as peptide antigens. To prepare complex antigen, cysteine was added to C-terminals of SVMP-pep-1 and SVMP-pep-2.

**
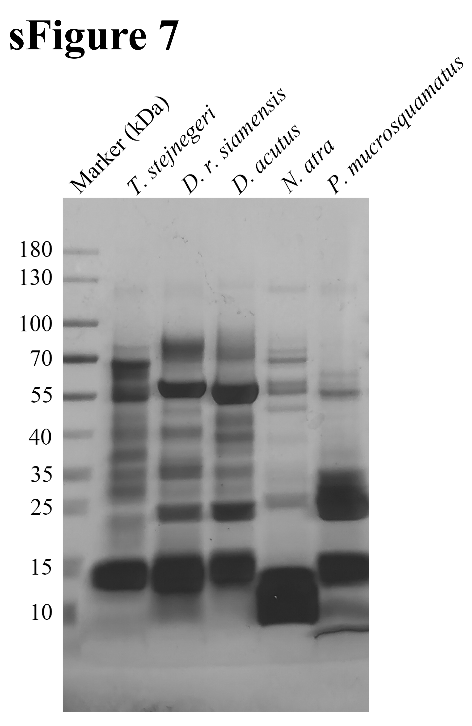
**

**Supplementary Figure 7 Coomassie brilliant blue staining of venom of five snake species with reducing SDS-PAGE.** From left to right, lanes are markers, *T. stejnegeri*, *D. r. siamensis*, *D. acutus*, *N. atra*, and *P. mucrosquamatus* venom.

**
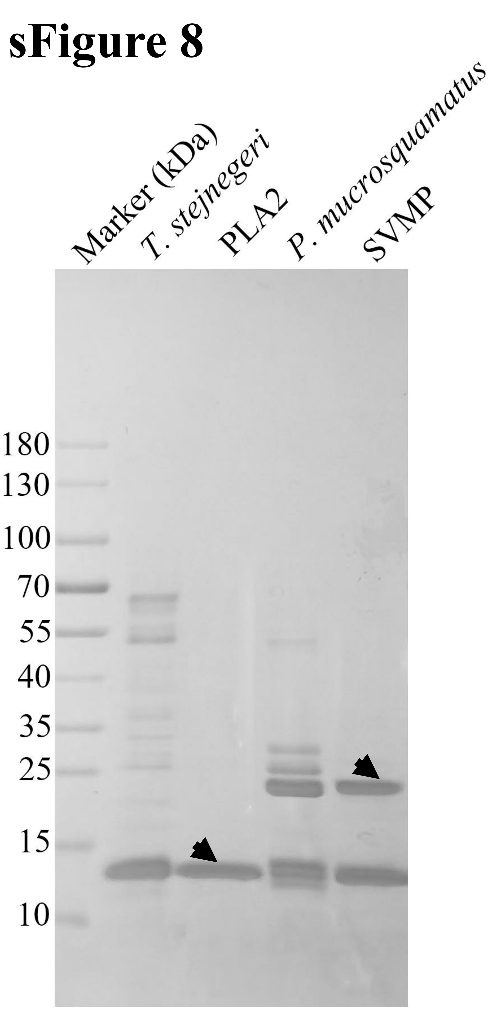
**

**Supplementary Figure 8 Reducing SDS-PAGE analysis of purified PLA_2_ and SVMP.** From left to right, lanes are markers, crude venom of *T. stejnegeri* and purified PLA_2_, crude venom of *P. mucrosquamatus* and purified SVMP. PLA_2_ and SVMP are indicated by black arrows.

**
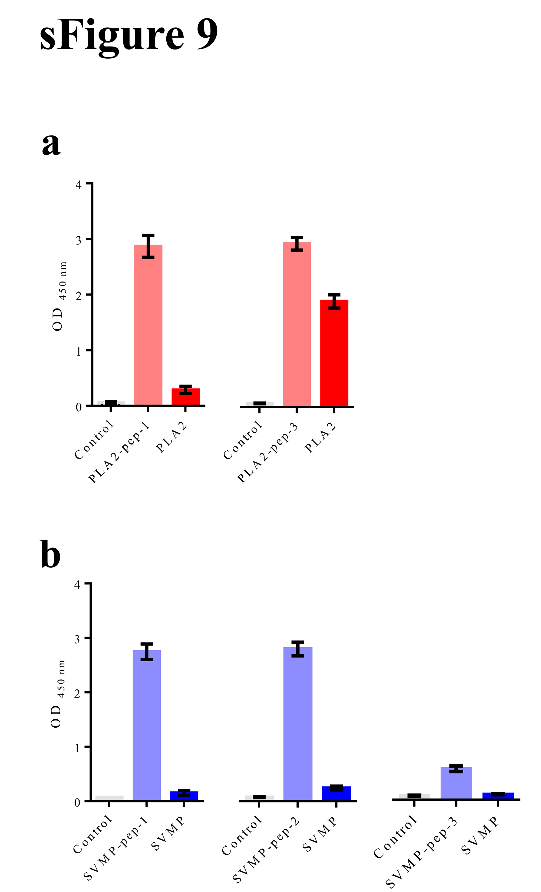
**

**Supplementary Figure 9 Binding of polyclonal antibodies against peptide antigens (pAbs-p) with peptide antigens and natural protein antigens.** Antibody recognition intensity between pAbs-p of anti-peptide, peptide, and natural protein antigens was detected by ELISA. Peptide and protein antigens (100 μl of antigen (10 μM) per well) were coated on an amino-coupling 96-well microplates (Corning Costar 2506, USA), blocked with BSA, incubated with primary antibody and anti-rabbit IgG secondary antibody, and added to HRP substrate. (**a**) pAbs-p against PLA2-pep-1 can bind to PLA 2-pep-1 and PLA_2_ protein, and recognition intensity to PLA2-pep-1 was stronger than that to PLA_2_ protein. Compared with PLA2-pep-1, pAbs-p against PLA2-pep-3 binding to PLA2-pep-3 and PLA_2_ protein is different, showed little difference in antibody recognition intensity. (**b**) pAbs-p against SVMP-pep-1, SVMP-pep-2, and SVMP-pep-3 can bind to peptide antigens and SVMP protein, with recognition intensity to peptides stronger than that to SVMP protein**.**


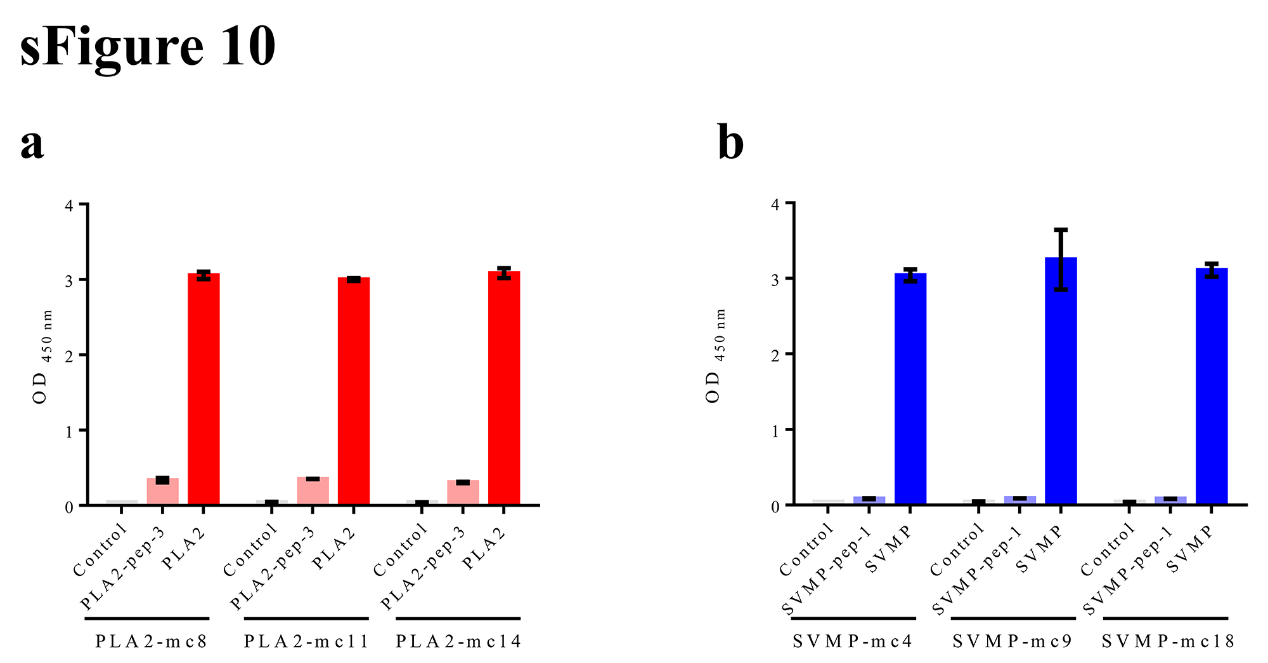


**Supplementary Figure 10 Binding of mAbs-n against natural PLA_2_ and SVMP proteins to peptide and natural protein antigens.** Antibody recognition intensity of mAbs-n against PLA_2_ and SVMP proteins to peptide and natural protein antigens was detected by ELISA. Peptide and protein antigens (100 μl, 10 μM per well) were coated on amino-coupling 96-well microplates (Corning costar 2506, USA), blocked with BSA, incubated with primary antibody (1 mg/ml, diluted 3 000 times) and anti-mouse IgG secondary antibody, and added to HRP substrate. (**a**) Anti-PLA_2_ mAbs-n could bind to PLA2-pep-3 and natural PLA_2_ protein, but recognition intensity to PLA_2_ was significantly stronger than that to peptide PLA2-pep-3 antigen. (**b**) Similarly, anti-SVMP mAbs-n could bind to SVMP-pep-1 and SVMP, and recognition intensity to SVMP was significantly stronger than that to peptide antigen SVMP-pep-1.

**
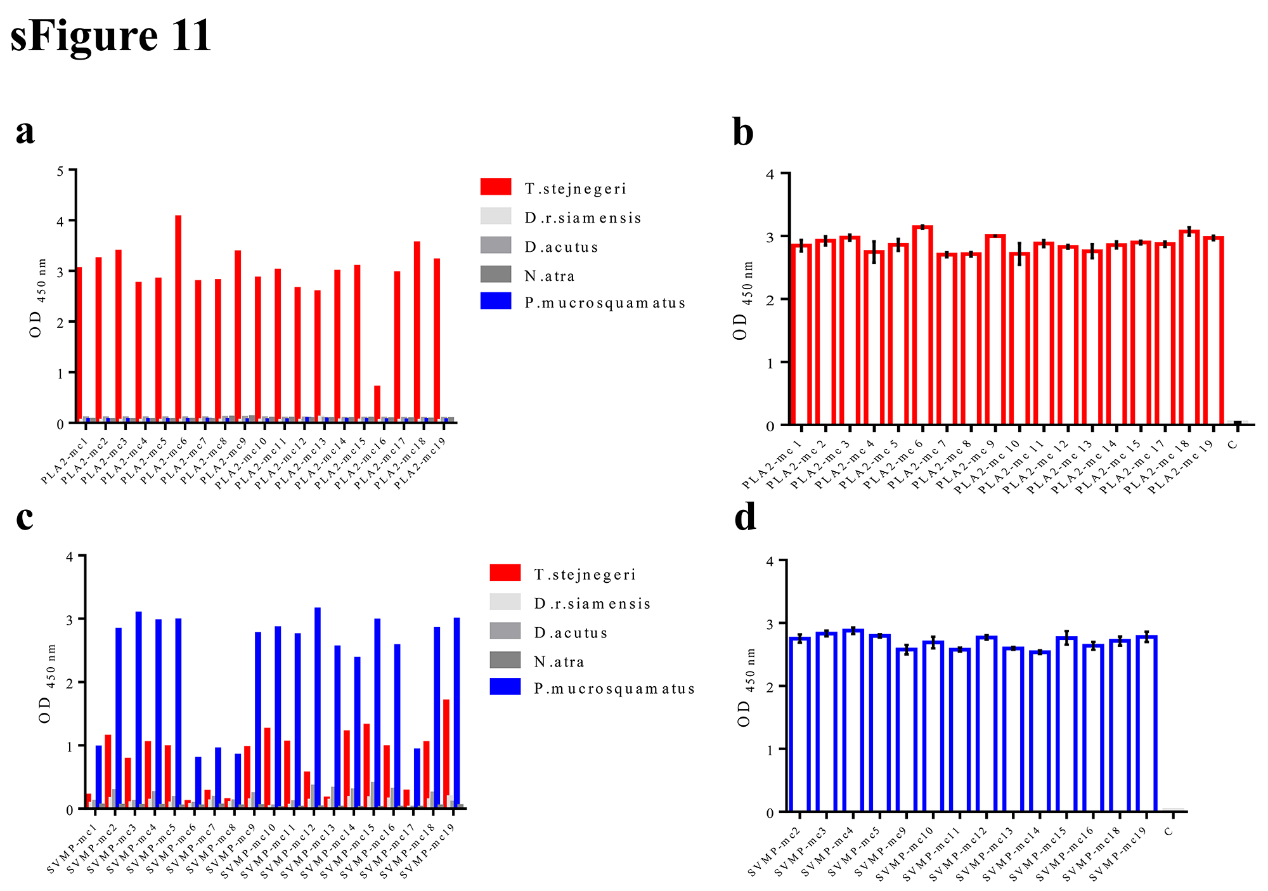
**

**Supplementary Figure 11 Test of specificity of mAbs-n to snake venom and** Antibody recognition intensity **of mAbs-n to natural PLA_2_ and SVMP protein antigens.** Antibody recognition intensity between mAbs-n of anti-natural protein antigens and crude venom and natural protein antigens tested by ELISA. Crude venom (100 μl, 10 μg/ml per pore) and PLA_2_ and SVMP protein antigens (100 μl, 1 μg/ ml per well) were coated on amino-coupling 96-well microplates (Corning Costar 2506), blocked with BSA, incubated with primary antibodies (1 mg/ml, diluted 3 000 times) and anti-mouse IgG secondary antibodies, and added to HRP substrate. Except for mAb-n mc-16, mAbs-n mc1-mc19 against PLA_2_ were specific to *T. stejnegeri* with high affinity (**a**). Except for mc-1, mc-6, mc-7, mc-8, and mc-17, all other anti-SVMP mAbs-n showed high recognition intensity to *P. mucrosquamatus* and weak reactions with *T. stejnegeri* and several other snake venoms (**c**). mAbs-n with strong recognition intensity were selected and their interactions with PLA_2_ (**b**) and SVMP proteins (**d**) were detected.

**
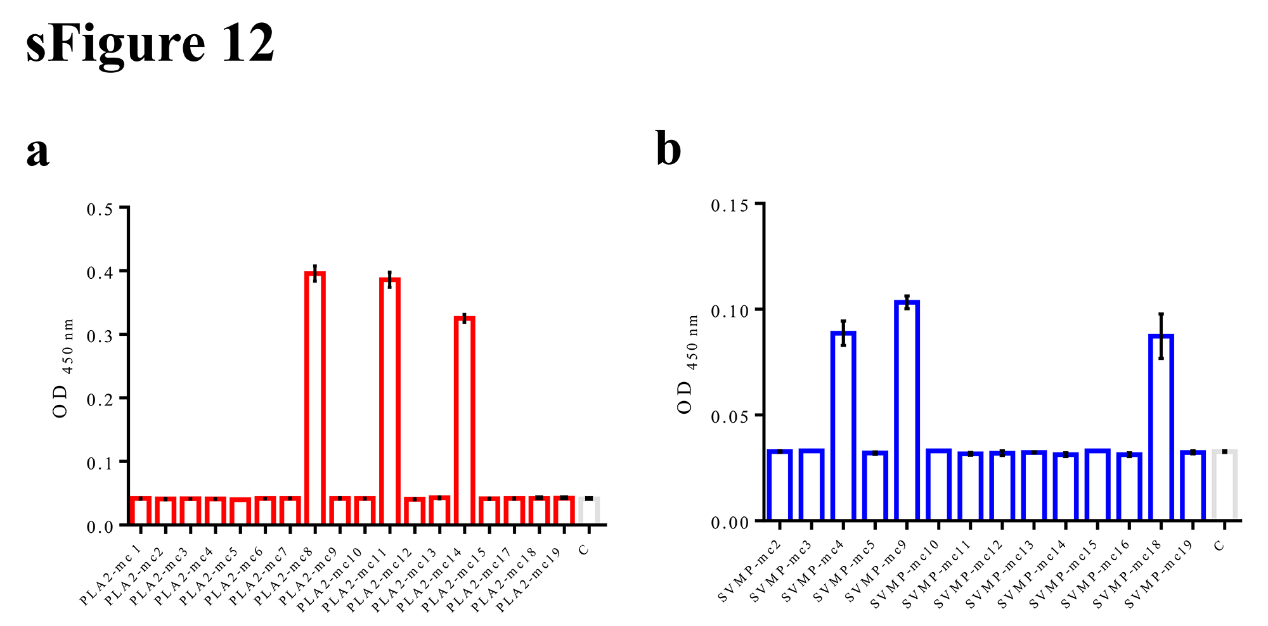
**

**Supplementary Figure 12 Screening of mAbs-n against peptide epitopes.** mAbs-n with strong recognition intensity (**Supplementary Figure 11**) were selected for peptide epitope screening by ELISA. Peptide antigens (100 μl, 10 μg/ml per well) were coated on amino-coupling 96-well microplates (Corning Costar 2506, USA), blocked with BSA, incubated with primary antibody (1 mg/ml, diluted 3 000 times) and anti-mouse IgG secondary antibody, and added to HRP substrate. Considering species specificity and surface accessibility of peptide antigens, peptide antigens PLA2-pep-3 from PLA_2_ and SVMP-pep-1 from SVMP were screened. Three mAbs-n against PLA2-pep-3 (**a**) and three mAbs-n against SVMP-pep-1 (**b**) were positive.

**
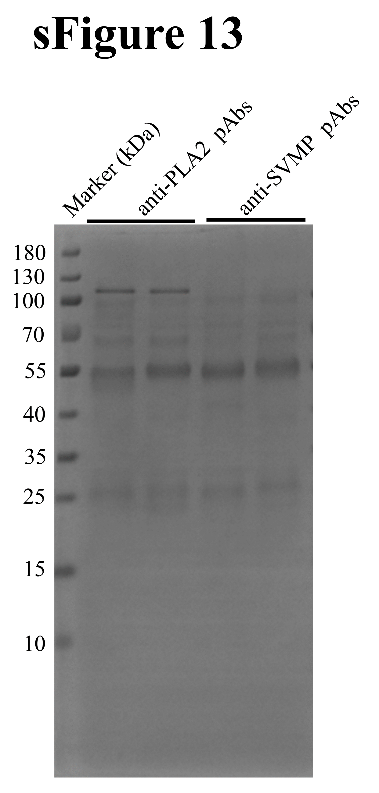
**

**Supplementary Figure 13 SDS-PAGE of purified pAbs-n against PLA_2_/SVMP.** pAbs-n were purified by Sepharose-protein A column and analyzed by reducing SDS-PAGE. Lanes from left to right are protein markers, anti-PLA_2_ pAbs-n, and anti-SVMP pAbs-n, respectively.


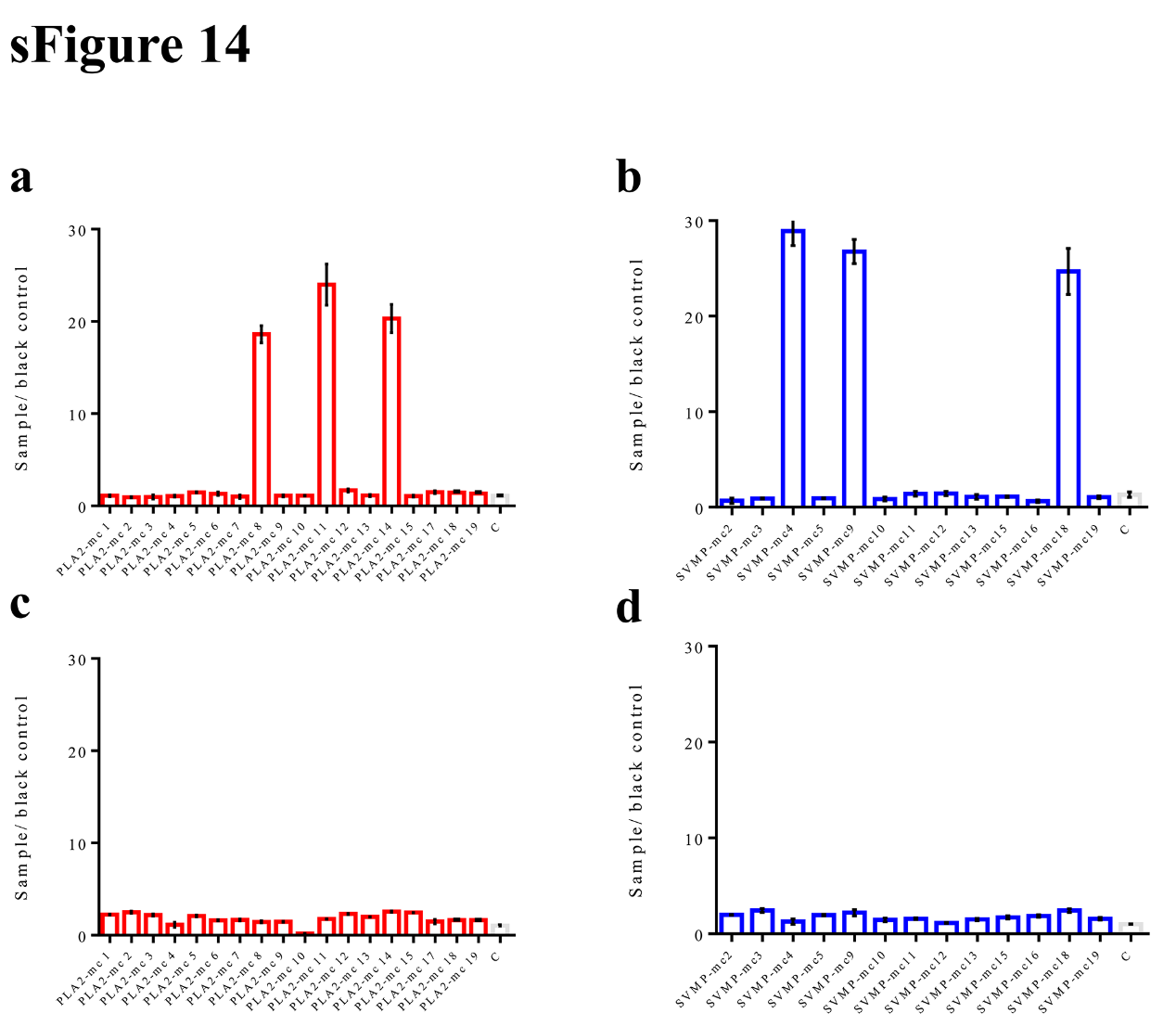


**Supplementary Figure 14 Matching mAbs-n and pAbs-n.** mAbs-n and pAbs-n against PLA_2_ and SVMP were matched according to their affinity to peptide antigens by ELISA. The mAbs-n and pAbs-n (100 μl, 10 μg/ml per well) were coated on amino-coupling 96-well microplates (Corning Costar 2506, USA). Plates were blocked with BSA, incubated with PLA_2_/SVMP (100 ng/ml) and HRP-labeled detector mAbs-n or pAbs-n (1 mg/ml, diluted 3 000 times), and added to HRP substrate. Through recognition intensity screening (**Supplementary Figure 10**), mAbs-n with strong binding ability were selected for matching. The best way of matching was that the polyclonal antibodies was coated on the plate with monoclonal antibodies as detector antibodies as reads at 450 nm and the sample/blank ratio were high for both **PLA_2_** (**a**) and **SVMP** (**b**). Successfully matched mAbs-n were antibodies against peptide epitopes PLA2-pep-3 and SVMP-pep-1. However, when coated with mAbs-n and using pAbs-n as detector antibodies, the effect was not good enough for detection of PLA_2_ and SVMP as reads at 450 nm and sample/blank ratios were lower for both PLA_2_ (**c**) and SVMP (**d**).

**
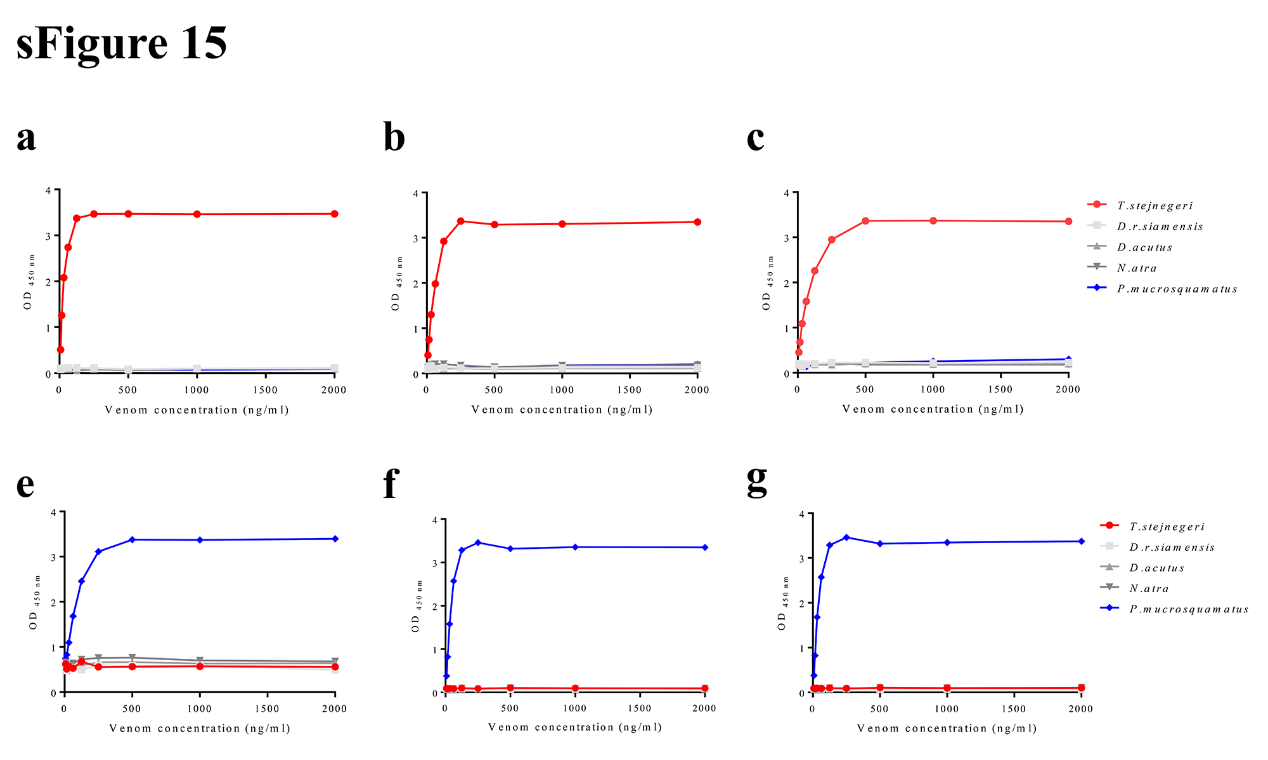
**

**Supplementary Figure 15 Snake venom testing after successful matching.** Venom from five snake species was tested and specificities of matched antibodies were verified by ELISA. pAbs-n were well matched with anti-PLA_2_ mAbs-n PLA2-mc8 (**a**), PLA2-mc11 (**b**), and PLA2-mc14 (**c**). PLA2-mc8 was the best matched antibody with the lowest detection line; pAbs-n were well matched with anti-SVMP mAbs-n SVMP-mc4 (**e**), SVMP-mc9 (**f**), and SVMP-mc18 (**g**). SVMP-mc18 was the best matched antibody with the lowest detection line.


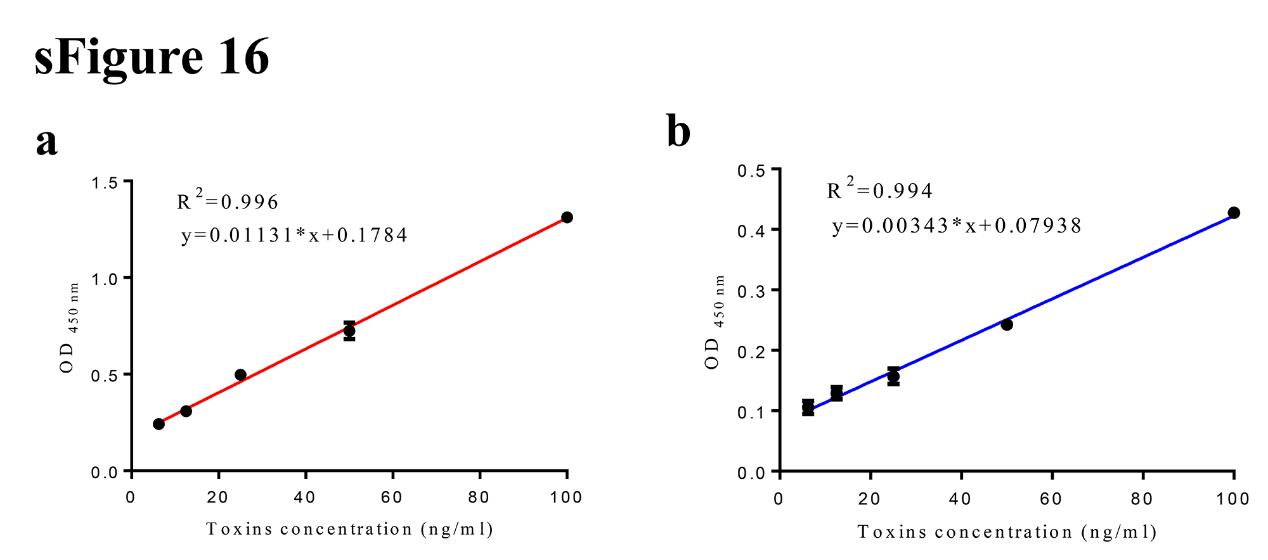


**Supplementary Figure 16 Standard curves for** **determination of concentrations of *T. stejnegeri* and *P. mucrosquamatus* venom.** Serially diluted venom proteins from *T. stejnegeri* (**a**) and *P*. *mucrosquamatus* (**b**) were subjected to PLA_2_-Ab-based or SVMP-Ab-based sandwich ELISA in triplicate. Standard curves were obtained by linear fitting in GraphPad Prism 6 software for determination of venom concentrations in simulated snakebite models.


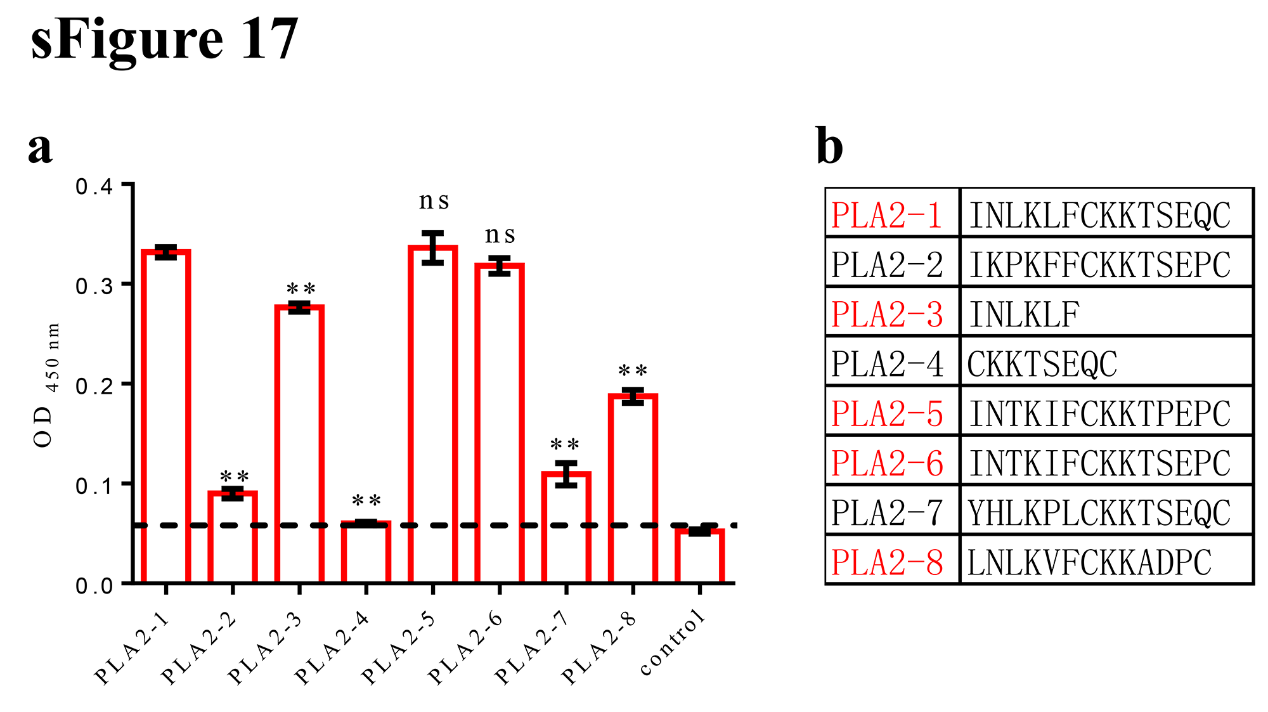


**Supplementary Figure 17 Anti-PLA2-pep-3 mAbs-n bind to other homologous sequences of PLA2-pep-3 from other species of *Trimeresurus*.** In sequence alignment, sequences derived from PLA_2_ from other species of *Trimeresurus* were highly homologous to PLA2-pep-3 (**Supplementary Figure 1**). Homologous sequences were synthesized and binding of anti-PLA2-pep-3 mAbs-n to homologous sequences was tested by indirect ELISA. The antibody binds strongly to PLA2-5 (INTKIFCKKTPEP) and PLA2-6(INTKIFCKKTSEPC), just as the antibody binds strongly to PLA2-1 (PLA2-pep-3).Antibody binds weakly to PLA2-2 (IKPKFFCKKTSEPC), PLA2-7 (YHLKPLCKKTSEQC), and PLA2-8 (LNLKVFCKKADPC). Binding epitope of anti-PLA2-pep-3 mAb was confirmed to be INLKLF by truncated PLA2-pep-3 polypeptide. ***P* < 0.01, significantly different compared with PLA2-1.


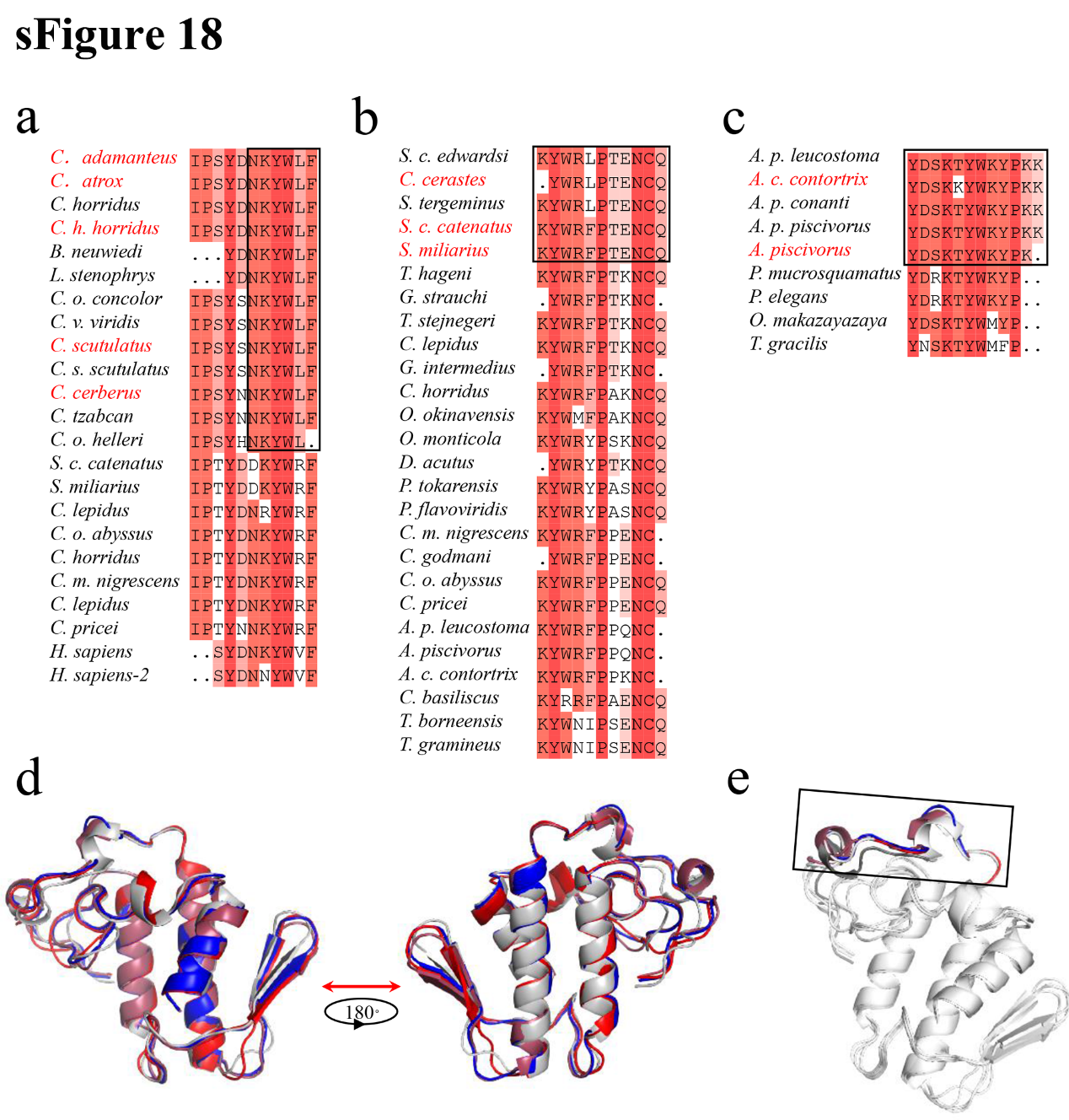


**Supplementary Figure 18 Diagnose GSAEPs of medically significant snakes in north American**  Through the analysis according the methods in this paper，three GSAEPs, including IPSYDNKYWLF, KYWRFPTENCQ and YDSKTYWKYPKK deriving from PLA2 of *C. atrox, S. miliarius* and *A. piscivorus* respectively, which are representing species of genus *Crotalus*, genus *Sistrurus* and genus *Agkistrodon* correspondingly, were found to identify genus *Crotalus* **(a)**, genus *Sistrurus* **(b)** and genus *Agkistrodon* **(c)**, respectively. The species marked in red are the main venomous snakes in north American, including 8 of totally 12 species of rattlesnakes (*C．adamanteus*, *C．atrox*, *C. h. horridus*, *C. scutulatus*, *C. Cerberus*, *C. cerastes*

, *S. c. catenatus* and *S. miliarius*) **(a and b)**, copperheads (*A. c. contortrix*) **(c)**, and cottonmouths (*A. piscivorus*) **(c)**. Highly conservative regions were marked by black box; 3D structure of the PLA2s got by homology modeling. Protein structure alignment results showed that the three GSAEPs were located in the same spatial position as PLA2-pep-3 of *T. stejnegeri* of genus *Trimeresurus, and* GSAEPs were highlighted by black box (**d and e**). PLA2 from *C. atrox*, *S. miliarius*, *A. piscivorus* and *T. stejnegeri*

were shown in red, pink, blue and black, respectively.


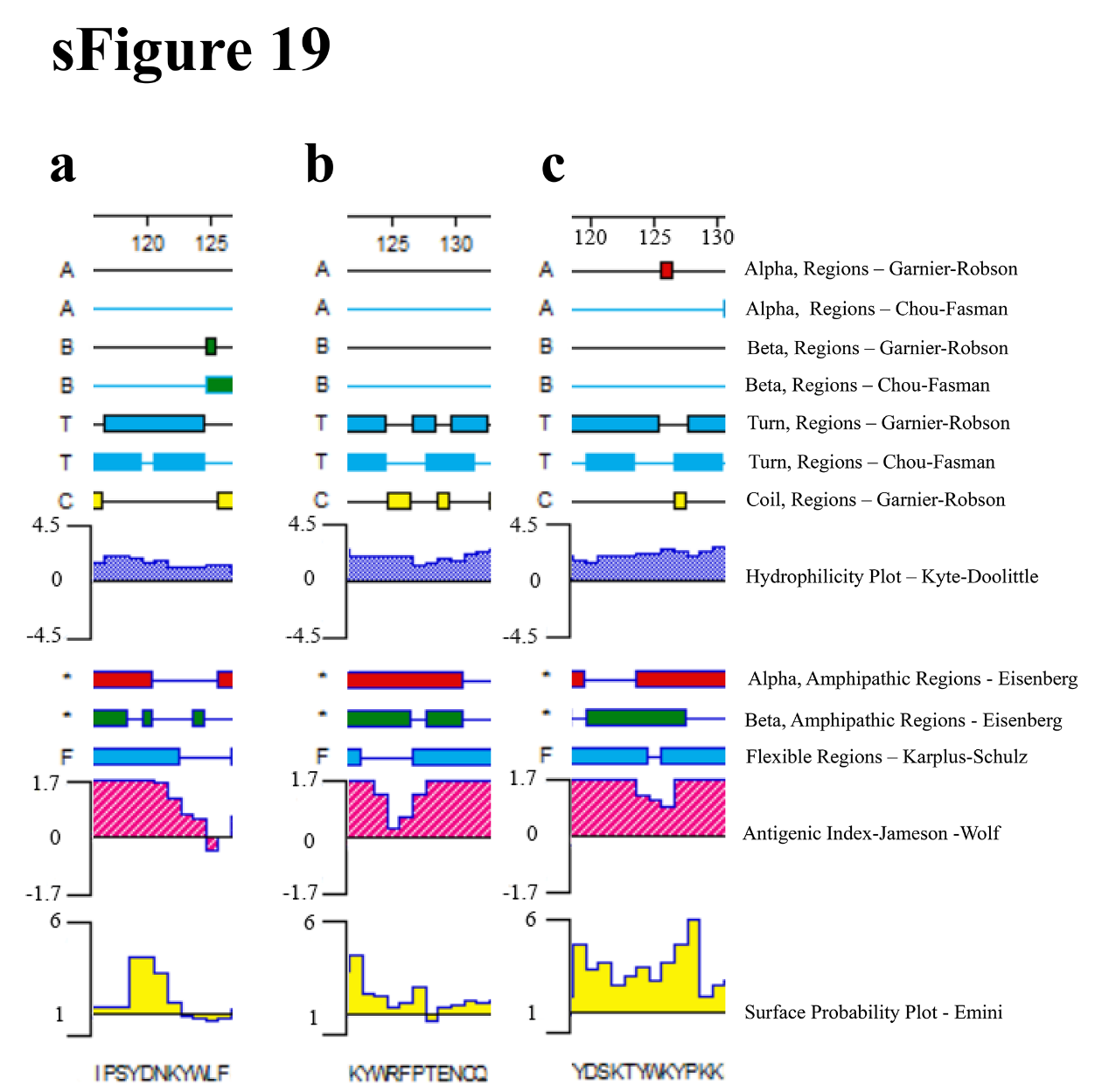


**Supplementary Figure 19 Antigenicity analysis of the three GSAEPs in Supplementary Figure 18** Protean module in Lasergene was used to analyze properties of IPSYDNKYWLF, KYWRFPTENCQ and YDSKTYWKYPKK, including secondary structure, hydrophilicity, immunogenicity, and spatial accessibility. The results shown that the three GSAEPs were hydrophilic, immunogenic, and surface-accessible.

**Supplementary Table 1. Summary of amino acid sequences of IgVH and IgVL sequences of mAbs-n.**
